# Prevalence and predictors of risk factors for non-communicable diseases in individuals aged 10–30 years: Findings from baseline cross-sectional assessment of V-CaN

**DOI:** 10.1371/journal.pgph.0005956

**Published:** 2026-10-30

**Authors:** Ashwini Kalantri, Mudita Joshi, Devyani Wanjari, Radhika Sharma, Anuj Mundra, Arjun Jakasania, Harshal Sathe, Sakshi Siriah, Pramod Bahulekar, Abhishek Raut, Chetna Maliye, Rameshwar J. Paradkar, Satish Kumar, Subodh S. Gupta, Bishan S. Garg

**Affiliations:** 1 Department of Community Medicine, MGIMS, Sewagram, Maharashtra, India; 2 Department of Psychiatry, MGIMS, Sewagram, Maharashtra, India; 3 Department of Biochemistry, MGIMS, Sewagram, Maharashtra, India; 4 Public Health Department, Wardha, Maharashtra, India; PLOS: Public Library of Science, UNITED STATES OF AMERICA

## Abstract

Adolescence and early adulthood are critical periods for establishing health behaviours that influence the future burden of non-communicable diseases (NCDs). The V-CaN trial aimed to develop, implement and evaluate a participatory health-promotion intervention using existing Village Health, Nutrition and Sanitation Committees (VHNSCs), women’s self-help groups (SHGs) and schools to address modifiable NCD risk factors among young people. The objective of this paper is to present the prevalence and predictors for behavioral, anthropometric and biochemical risk factors among young people of 10–30 years age group. The baseline assessment included a cross-sectional survey of 12,000 participants selected by simple random sampling from a Health and Demographic Surveillance System sampling frame. Prevalence of behavioural and anthropometric risk factors was assessed in the full sample, while biochemical risk factors were assessed in a subsample of 1,600 participants. Best-fit multivariable regression models were used to identify predictors of physical inactivity, stress, tobacco use and dietary behaviours. Mean daily salt and sugar consumption was 8.7 (±3.1) g and 44.7 (±18.2) g, respectively. Prevalence of smoking, smokeless tobacco use, alcohol consumption, inadequate physical activity, overweight and obesity was 0.3%, 15.3%, 1.8%, 32.4%, 8.1% and 7.6%, respectively. Moderate stress, high stress, anxiety and depression were reported by 74.0%, 4.5%, 16.0% and 27.5% of participants, respectively. Mean cholesterol levels were higher among 20–30 years participants (147.8 ± 33.9) as compared to the adolescents (130.0 ± 27.0). Age, education, occupation, marital status, alcohol and smokeless tobacco use, stress, anxiety, depression and elevated blood pressure were significantly associated with selected modifiable NCD risk factors. The findings demonstrate a substantial burden of modifiable NCD risk factors among young people, particularly smokeless tobacco use, inadequate physical activity and psychological distress. Strengthening prevention, early screening and health-promotion interventions through existing community and educational platforms could support timely risk reduction and contribute to long-term NCD prevention.

## Introduction

The burden of noncommunicable diseases (NCDs) is escalating in low- and middle-income nations like India, which are concurrently facing the challenges of improving nutrition, controlling infectious disease, and improving maternal and child health services. Over the next 25 years, there will likely be a large increase in the burden of cancer, diabetes, chronic respiratory diseases, cardiovascular diseases, and mental disorders. By 2030, NCDs are predicted to be responsible for around 75% deaths in India [1]. According to the National Family Health Survey-5 (NFHS-5), the overall prevalence of Diabetes Mellitus (DM) in females and men is 2.9% and 4.2%, respectively, whereas the prevalence of hypertension (HTN) in females and males is 13.5% and 21.6%, respectively [2]. Several studies have shown that early interventions targeting modifiable risk factors for NCDs are necessary for both primary and secondary prevention of NCD [3,4]. The Comprehensive National Nutrition Survey (CNNS) data from 2016–2018 for all 29 Indian states reveals a startlingly high prevalence of HTN in Indian youth: 35% of children aged 10–12 and 25% of those aged 13–19 had blood pressure in the stage 1 or 2 hypertension range, as defined by the American Academy of Paediatrics cut points from 2017. In addition, obesity, high blood sugar, and lipid abnormalities were among the major cardio-vascular disease (CVD) risk factors that were more prevalent in young people with high blood pressure [5].

Evidence from low- and middle-income nations suggest that community-based interventions through health promotion programs can reduce the prevalence of risk factors for NCDs. Such interventions greatly lower the hazards associated with changing one’s diet and lifestyle, drinking alcohol, smoking, and other habits [4]. 9.3% of Indian adults living in rural areas have been observed to have metabolic syndrome, which is characterized by a clustering of abdominal obesity, hypertension, impaired glucose metabolism, and dyslipidemia [6]. According to a meta-analysis, the prevalence of childhood obesity in India is 8.4% and that of childhood overweight is 12.4% [7]. According to the National non-communicable disease monitoring survey the prevalence of adolescent overweight and obesity is 6.2% and 1.8% respectively. Around 25% adolescents have inadequate physical activity and approximately 3% consume tobacco daily [8]. In Wardha, 4.3% of kids are either overweight or obese, while 57% of teenage girls have dietary deficiencies [9].

Adolescence and early adulthood are pivotal periods for the establishment of health behaviors with long-term impact on the incidence of NCDs such as the mean age for initiation of consuming smokeless tobacco which was reported as 9.9 years in India as per Global Youth Tobacco Survey-4 (GYTS) [10]. For the understanding and primary prevention of such modifiable risk factors during these formative years can significantly reduce the future burden of NCDs. This necessitates a comprehensive risk factor profiling in the age group of 10–30 years is not only vital for immediate health benefits but also for curbing the long-term prevalence of NCDs. Community engagement for primary prevention of NCDs is important and the same has also been emphasized in the National Programme for Non-communicable Diseases in India [11,12].

We therefore, planned a hybrid implementation/ effectiveness trial to develop community-based strategies for primary prevention of NCDs and assess the effectiveness and implementation outcomes of these strategies [13]. The current paper presents the findings from the baseline cross-sectional survey and reports the estimates of the behavioral (dietary habits, obesity, tobacco use, stress, anxiety, depression, alcohol consumption, physical inactivity) and biochemical (high blood pressure, deranged lipid panel and high blood sugar) modifiable risk factors for NCDs. Beyond such prevalence estimates reported in various national surveys, this study also reports the predictors of these common modifiable risk factors in individuals aged 10–30 years.

## Materials and methods

### Study setting

This study was conducted as a part of Vitalizing Community for Health Promotion Against Modifiable Risk Factors of Noncommunicable Diseases (V-CaN) trial (a hybrid type 2 implementation effectiveness trial) implemented in Wardha district, Maharashtra, India. This trial was registered under the Clinical Trials Registration India CTRI/2020/10/028700; https://ctri.nic.in/Clinicaltrials/showallp.php?mid1=47597&EncHid=&userName=V-CaN (International Registered Report Identifier (IRRID): DERR1-10.2196/42450) on October 28, 2020. Ethics statement: Ethics approval was obtained from the institutional ethics committee, before the start of the trial vide letter MGIMS/IEC/COMMED/79/2020. During the study, the data enumerator verbally explained about the study to the participants and asked them to sign the informed consent form. For adolesecnt participants, written consent was taken from either of the parent and verbal assent was taken from the participants. The response of the participants was also documented as a part of the questionnaire. Additionally, consent was also taken from the subset identified for biochemical investigations. The detailed protocol of the V-CaN trial has been published elsewhere [13].

### Study population

The study population consisted of all individuals aged 10–30 years residing in the study area and enrolled in our Health and Demographic Surveillance System (HDSS).

### Sample size and sampling

Sample size was estimated to assess the effectiveness of our interventions under the V-CaN trial assuming the lowest prevalence of NCD-related behavioral risk factors at 13% [6–8,28,29] to detect a 25% reduction in the risk factors, with 95% confidence, 80% power, intra-cluster correlation coefficient of 2, an approximate sample size was calculated to be of 12,000 participants. For biochemical risk factors, we derived a sample size of 1600 to detect a 10% reduction in the mean values at 90% power and 95% confidence levels and accounting for 10% sample wastage. The baseline assessment for behavioral and biochemical risk factors was carried out in all the 12,000 and 1600 respondents respectively. In the present manuscript, we present the findings of this baseline assessment.

To select the respondents, a sampling frame was drawn from the already existing database under the HDSS for the study area. Stratified random sampling (stratified for sex) was performed for identifying the respondents for behavioral risk factors. A list of all those who consented for blood samples for estimation of biochemical risk factors was prepared immediately after this initial behavioral risk factor assessment. From this list the required 1600 respondents for biochemical risk factor estimation were selected using the “sample_n” function of the “dplyr” package in R software (version 4.1.2; R Foundation for Statistical Computing). The flowchart in Fig 1 provides details on the sample collection.

**Fig 1:**
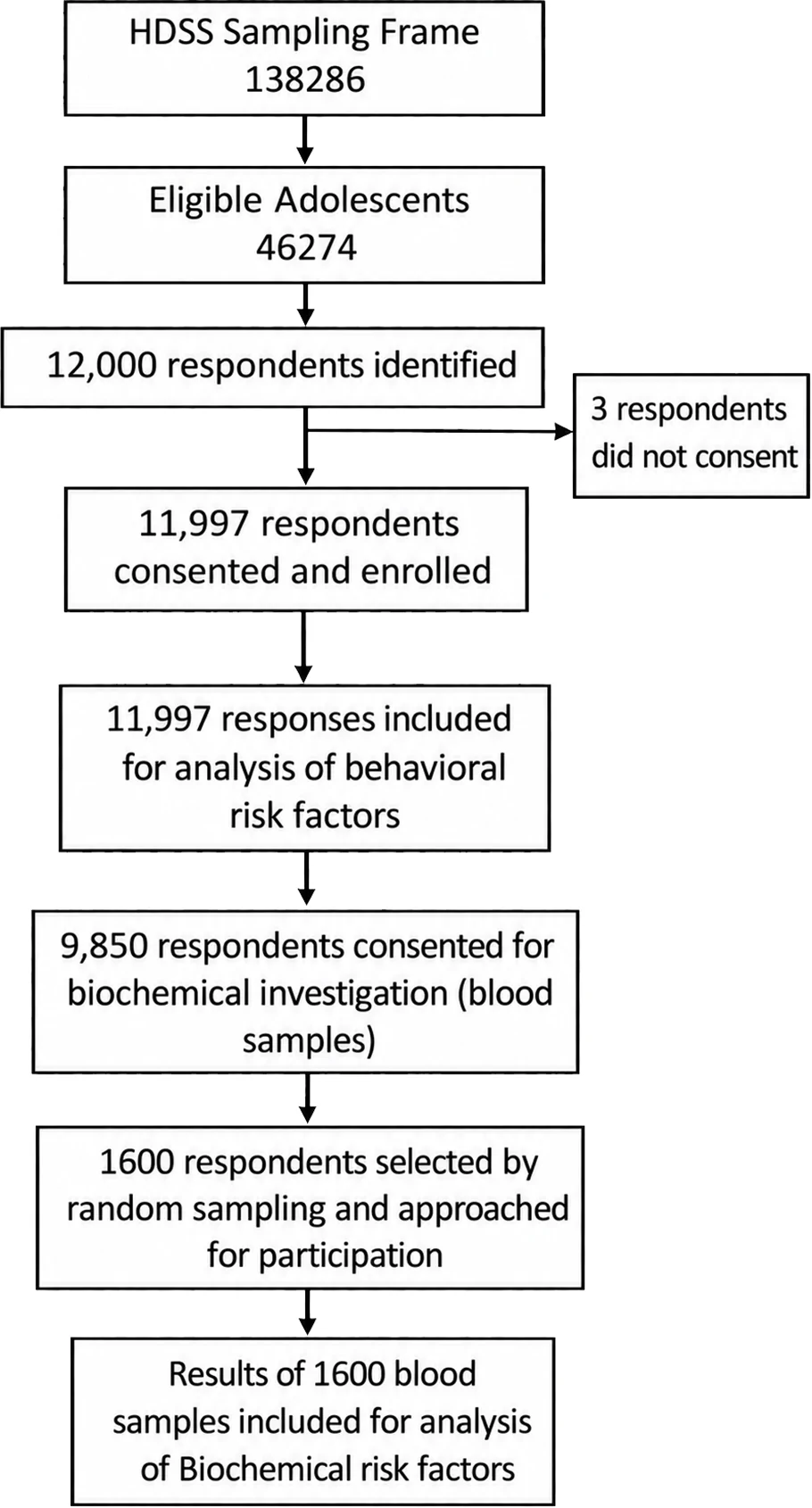
Participant flow.

### Data collection and analysis

The baseline data was collected using the Survey Solutions software [14] by trained data enumerators. The data was cleaned and analyzed using R version 4.4.0 using packages “dplyr”, “lubridate”, “stringr”, “tidyr” and “anthroplus” [15]. All of the data being reported here was collected as a part of the survey except their economic status. The information on their economic status was extracted from the database of the most recent round of the HDSS conducted in January 2023. The Wealth index was constructed using principal component analysis. For missing data, observations were removed from the analysis and for extreme outliers, data was imputed using median of the variable. Categorical variables have been summarized using frequency and percentage. Continuous variables have been summarized using mean (SD) or median (IQR). Multivariate ordinal regression was performed to depict the predictors of stress. For studying the predictors of other common modifiable risk factors (smokeless tobacco consumption, physical activity, daily salt and sugar consumption) logistic regression was performed. The operational definitions are stated in Table 1.

**Table 1:** Operational definitions.

Behavioral risk factors	Variables	Approach for estimation	Operational definitions
Dietary practices (WHO)	Inadequate fruits and vegetables intake	The number of fruits and vegetable servings was asked to the participant.	< 5 servings per person per day [16]
	Excess salt intake	For calculating the sugar and salt consumption per person per day, we divided the total salt and sugar intake with the number of family members as well as the days required to consume the packet completely.	> 5 grams per person per day [17]
	Excess sugar intake		> 50 grams per person per day [18]
Physical activity (Global physical activity questionnaire)	Inadequate physical activity	The questions from Global physical activity questionnaire were utilised as a part of the interview questionnaire and analysed based on the analysis guide for the questionnaire.	< 600 met-minutes per person per day [19]
Obesity (WHO Southeast Asian classification) [20]	Severe Underweight	Body Mass Index (BMI) was calculated based on respondents height and weight measured during the survey and classified based on WHO Southeast Asian classification.	BMI < 16.5
	Underweight		BMI 16.5 to 18.5
	Normal		BMI 18.5 to 22.9
	Overweight		BMI 22.9 to 24.9
	Obese		BMI > 24.9
Addictions	Smokeless tobacco consumers	Participants were directly asked for their history of respective addictions as a part of the questionnaire.	Respondents who were consuming any form of smokeless tobacco like kharra, betel quid, chewing tobacco, etc were reported as consumers of smokeless tobacco.
	Current smokers		Respondents who reported smoking any tobacco products, such as cigarettes, bidis, cigars or pipes at the time of survey were considered as current smokers.
	Alcohol consumers (Past 30 days)		Respondents consuming any alcohol in 30 days preceding the survey have been considered as alcohol consumers in past 30 days.
Hypertension (HTN)	Normotensive	Blood pressure was measured three times for all individuals during the survey and the average of these three readings was considered for analysis.	This categorization was done using reference tables and cut-offs as suggested by the American Academy of Pediatrics [21]
	Pre-hypertensive		
	Hypertensive		
Diabetes (DM)	Normal Glucose levels	Blood glucose levels were tested at the time of the survey and the readings were collected. Based on the standard cut-offs, the participants were categorised in the three categories.	*≤ 110 mg/dl*
	Pre-diabetic		110 mg/dl to 126 mg/dl
	Diabetic		> 126 mg/dl
Mental health parameters	Stress (Perceived Stress Score (PSS))	To measure the perceived stress severity, we used the Perceived Stress Score and analysed it based on the cut-offs for PSS [22,23].	Low Perceived Stress: Those with PSS score between 0 and 13
			Moderate Perceived Stress: Those with PSS score between 14 and 26
			High Perceived Stress: Those with PSS score between 27 and 40
	Anxiety (Patient health questionnaire- 4 (PHQ-4))	The 4 questions from PHQ-4 were asked to all the respondents and were interpreted based on the recommended scoring and interpretation of PHQ-4.	Those with a score of 3 or more in the first two questions of PHQ-4 were reported to have anxiety [24].
	Depression (PHQ- 4)		Those with a score of 3 or more in the last two questions of PHQ-4 were reported to have anxiety [24].

After considering the variables based on theory of change for the project as well as variables with p values of <0.2 on bivariate analysis [13], we employed a forced entry method to add these variables to the model. After assessing carefully for multicollinearity based on the Variance Inflation factor (VIF) and tolerance values, the variables showing moderate to high collinearity have been omitted from the analysis. After assessing multicollinearity and other model parameters like Akaike Information Criterion (AIC), Bayesian Information Criterion (BIC), and R-squared values among the candidate models, we have reported the best fitting model. Models were created with some of the common risk factors of NCDs as outcome variables- stress, smokeless tobacco consumption, physical inactivity, excess salt and sugar consumption.

## Results

The socio-demographic characteristics and risk factors have been summarized separately for the two age groups as specified earlier. Regarding the education status of the participants, 35.4% of the respondents had no or primary education whereas 12.1% had completed graduation and above. 59.5% of the respondents were students. The data on economic characteristics was available for only 9862 participants through our Health and Demographic Surveillance System (HDSS) which has been summarized as wealth quintiles. Majority of the respondents belonged to the fifth (23.6%) and fourth (23.5%) quintile of wealth index. Nearly one-fifth (20.8%) of the respondents were married at the time of data collection. Almost half (49.5%) of the respondents belonged to the Other Backward Class (OBC) category followed by Scheduled Tribe (ST) (18.7%), Scheduled Caste (SC) (15.4%), Vimukt Jati/Nomadic Tribes (VJ/NT) (12.3%) and Open (4.2%). The median (Inter quartile range) years of schooling was 11 (8, 12) years. (Table 2)

**Table 2:** Description of socio-demographic characteristics of the respondents.

Characteristic (N)		10-19 years n(%)	20-30 years n(%)	Overall n(%)
***Sex (N = 11,997)***	Female	2,816 (50.1%)	3,094 (48.6%)	5,910 (49.3%)
	Male	2,809 (49.9%)	3,278 (51.4%)	6,087 (50.7%)
***Education (N = 11,991)***	No or primary education	3,247 (57.8%)	994 (15.6%)	4,241 (35.4%)
	Secondary	1,401 (24.9%)	1,336 (21.0%)	2,737 (22.8%)
	Higher Secondary	945 (16.8%)	2,615 (41.0%)	3,560 (29.7%)
	Graduate and above	26 (0.5%)	1,427 (22.4%)	1,453 (12.1%)
***Occupation (N = 11,997)***	Farmer	13 (0.2%)	430 (6.7%)	443 (3.7%)
	Homemaker	25 (0.4%)	1,637 (25.7%)	1,662 (13.9%)
	Labourer	89 (1.6%)	1,579 (24.8%)	1,668 (13.9%)
	Service	11 (0.2%)	773 (12.1%)	784 (6.5%)
	Student	5,436 (96.6%)	1,704 (26.7%)	7,140 (59.5%)
	Unemployed	51 (0.9%)	249 (3.9%)	300 (2.5%)
***Wealth quintiles (N = 9,862)***	Lowest	600 (12.9%)	551 (10.6%)	1,151 (11.7%)
	Second	1,019 (21.9%)	913 (17.5%)	1,932 (19.6%)
	Middle	1,011 (21.7%)	1,120 (21.5%)	2,131 (21.6%)
	Fourth	1,078 (23.2%)	1,242 (23.8%)	2,320 (23.5%)
	Highest	941 (20.2%)	1,387 (26.6%)	2,328 (23.6%)
***Marital status (N = 11,997)***	Currently married	27 (0.5%)	2,465 (38.7%)	2,492 (20.8%)
	Divorced/ Separated	4 (0.1%)	43 (0.7%)	47 (0.4%)
	Never married	5,594 (99.4%)	3,864 (60.6%)	9,458 (78.8%)
***Caste (N = 11,997)***	Open	240 (4.3%)	260 (4.1%)	500 (4.2%)
	OBC	2,758 (49.0%)	3,177 (49.9%)	5,935 (49.5%)
	SC	887 (15.8%)	962 (15.1%)	1,849 (15.4%)
	ST	1,054 (18.7%)	1,188 (18.6%)	2,242 (18.7%)
	VJ/NT/Others	686 (12.2%)	785 (12.3%)	1,471 (12.3%)
Years of schooling (11,997)*		9 (7.0, 11)	12 (10.0, 14)	11 (8.0, 12)

*median (IQR) has been reported for years of schooling.

OBC – Other Backward Class, SC- Scheduled caste, ST- Scheduled tribe, VJ– Vimukt Jati, NT- Nomadic Tribes, IQR- Inter quartile range.

We have reported the prevalence of common risk factors of non-communicable diseases such as- dietary practices, addiction, inadequate physical activity, mental health status (perceived stress, anxiety and depression), body mass index (BMI), hypertension, diabetes status, addictions like tobacco and alcohol consumption (Table 3a) and the biochemical measurements (Lipid panel and blood glucose) for a subgroup of 1600 respondents (Table 3b). Among dietary practices, we have reported servings of fruits and vegetables along with daily dietary intake of salt and sugar. Based on our observations, 99.9% consumed less than 5 servings of fruits and vegetables. 95.9% of the respondents consume more than 5 grams salt per person per day which exceeds the recommended intake of 5 grams per person per day whereas 27.4% respondents consumed more sugar than the recommended limit of 50 grams.

**Table 3:** a. Prevalence of behavioral risk factors for NCDs. b. Distribution of biochemical risk factors.

Characteristic (N = 11,997)		10-19 years n(%)	20-30 years n(%)	Overall n(%)
***Fruit and vegetable servings per day (N = 11,986)***	≥ 5 servings per day	5 (0.1%)	1 (0.0%)	6 (0.1%)
	< 5 servings per day	5,616 (99.9%)	6,364 (100.0%)	11,980 (99.9%)
***Salt consumption (grams per person per day)***	≤ 5 grams	216 (3.8%)	270 (4.2%)	486 (4.1%)
	> 5 grams	5,409 (96.2%)	6,102 (95.8%)	11,511 (95.9%)
***Sugar consumption (grams per person per day)***	≤ 50 grams	4,269 (75.9%)	4,440 (69.7%)	8,709 (72.6%)
	> 50 grams	1,356 (24.1%)	1,932 (30.3%)	3,288 (27.4%)
***Body mass index (N = 11,798)***	Severe Underweight	413 (7.4%)	484 (7.8%)	897 (7.6%)
	Underweight	997 (17.8%)	1,274 (20.6%)	2,271 (19.2%)
	Normal	3,825 (68.1%)	2,952 (47.7%)	6,777 (57.4%)
	Overweight	290 (5.2%)	662 (10.7%)	952 (8.1%)
	Obese	90 (1.6%)	811 (13.1%)	901 (7.6%)
***Hypertension (N = 11,995)***	Normal	4,085 (72.6%)	3,709 (58.2%)	7,794 (65.0%)
	Elevated	460 (8.2%)	565 (8.9%)	1,025 (8.5%)
	HTN1	1,012 (18.0%)	1,890 (29.7%)	2,902 (24.2%)
	HTN2	66 (1.2%)	208 (3.3%)	274 (2.3%)
***Diabetes Mellitus (N = 1,599)***	Normal	751 (97.8%)	753 (90.6%)	1,504 (94.1%)
	Pre-diabetic	14 (1.8%)	65 (7.8%)	79 (4.9%)
	Diabetic	3 (0.4%)	13 (1.6%)	16 (1.0%)
***Perceived stress***	Low	1,775 (31.6%)	805 (12.6%)	2,580 (21.5%)
	Moderate	3,730 (66.3%)	5,144 (80.7%)	8,874 (74.0%)
	High	120 (2.1%)	423 (6.6%)	543 (4.5%)
***Anxiety***		710 (12.6%)	1,207 (18.9%)	1,917 (16.0%)
***Depression***		1,006 (17.9%)	2,292 (36.0%)	3,298 (27.5%)
***Current smokers***		3 (0.1%)	37 (0.6%)	40 (0.3%)
***Smokeless tobacco consumers***		210 (3.7%)	1,630 (25.6%)	1,840 (15.3%)
***Alcohol consumers (past 30 days)***		5 (0.1%)	211 (3.3%)	216 (1.8%)
***Inadequate physical activity***		1,459 (25.9%)	2,433 (38.2%)	3,892 (32.4%)
**b. Distribution of biochemical risk factors**				
**Characteristic**		**10-19 years** N = 849	**20-30 years** N = 751	**Overall** N = 1600
***Glucose***^**1**^ ***(gm/dl)***		81.0 (12.6)	84.9 (19.4)	83.0 (16.6)
***Cholesterol***^**1**^ ***(mg/dl)***		130.0 (27.0)	147.8 (33.9)	139.2 (32.0)
***Triglycerides (TG)***^**2**^ ***(mg/dl)***		76.0 (59.0-98.7)	83.5 (61.0-113.0)	79.0 (60.0-106.0)
***Low-density Lipoprotein (LDL)***^**1**^ ***(mg/dl)***		71.6 (21.0)	84.1 (26.2)	78.1 (24.6)
***Very low-density Lipoprotein (VLDL)***^**2**^ ***(mg/dl)***		15.2 (11.8-19.7)	16.7 (12.2-22.6)	15.8 (12.0-21.2)
***High-density Lipoprotein (HDL)***^**1**^ ***(mg/dl)***		41.7 (9.8)	44.5 (11.0)	43.2 (10.5)

n (%) for categorical variables. ^***1***^Mean (SD); ^***2***^Median (25%-75%).

Most (57.4%) of the respondents had normal BMI, 19.2% were underweight whereas the prevalence of severe underweight, overweight and obesity was 7.6%, 8.1% and 7.6% respectively. The prevalence of overnutrition was seen to be higher in the 20–30 years age group. Nearly two-third respondents (65.0%) were normotensive, 24.2% were in HTN grade 1 and 2.3% fell in HTN grade 2. 94.1% had normal blood glucose levels, 4.9% were pre-diabetic and 1% were diabetic.

Higher levels of stress, anxiety and depression were seen among individuals aged 20–30 years. Overall 21.5% of the respondents had low stress, almost three-fourths (74.0%) had moderate stress and 4.5% had high stress levels. The overall prevalence of anxiety and depression was 16% and 27.5% respectively in the study population.

Only 0.3% of our respondents reported the practice of smoking, 15.3% reported consumption of smokeless tobacco and 1.8% reported alcohol consumption in 30 days preceding the survey. Around 32.4% turned out to be physically inactive with more physically inactive participants (38.8%) from the 20–30 age group.

Table 3b suggests prevalence of biochemical risk factors. Overall, mean (SD) glucose level was 83.0 (16.6) gm/dl. The mean (SD) levels of cholesterol, LDL, and HDL were 139.2 (32.0) mg/dl, 78.1 (24.6) mg/dl and 43.2 (10.5) mg/dl respectively. We are reporting the median values of TG and VLDL here due to non-normal distribution in the study sample. The Median (IQR) value of TG and VLDL were 79.0 (60.0 - 106.0) mg/dl and 15.8 (12.0 - 21.2) mg/dl.

As perceived stress was the most prevalent risk factor in our study population, we are reporting the association between stress with the socio-demographic variables and the risk factors under study before the other risk factors in Table 4. The best-fitting model for stress as an outcome was created after adjusting for variables mentioned in the footnote of Table 4. It was selected based on comparisons of the Akaike Information Criterion (AIC), Bayesian Information Criterion (BIC), and R-squared (Nagelkerke’s R² value = 0.455) values among the candidate models.

**Table 4:** Association of stress with socio-demographic and other behavioral characteristics among 10-30 years old persons.

Characteristics		Low stress	Moderate stress	High perceived stress	OR (95% CI)	aOR (95% CI)
***Age category***	10-19 years	1,775 (68.8%)	3,730 (42.0%)	120 (22.1%)	1 (ref)	1 (ref)
	20-30 years	805 (31.2%)	5,144 (58.0%)	423 (77.9%)	3.20 (2.93 - 3.49)	**1.82** **(1.60 - 2.06**)*
***Sex***	Female	639 (24.8%)	5,008 (56.4%)	263 (48.4%)	1 (ref)	1 (ref)
	Male	1,941 (75.2%)	3,866 (43.6%)	280 (51.6%)	0.34 (0.31 - 0.37)	0.37 (0.33-0.41)*
***Caste***	Open	70 (2.7%)	414 (4.7%)	16 (2.9%)	1 (ref)	1 (ref)
	OBC	1,287 (49.9%)	4,398 (49.6%)	250 (46.0%)	0.71 (0.57 - 0.88)	**0.77** (0.61 - 0.99)*
	SC	386 (15.0%)	1,374 (15.5%)	89 (16.4%)	0.76 (0.60 - 0.95)	0.82 (0.63 - 1.07)
	ST	502 (19.5%)	1,606 (18.1%)	134 (24.7%)	0.74 (0.59 - 0.92)	0.79 (0.61 - 1.03)
	VJ/NT/Others	335 (13.0%)	1,082 (12.2%)	54 (9.9%)	0.66 (0.52 - 0.83)	**0.64** (0.49 - 0.84)*
***Marital status***	Currently married	136 (5.3%)	2,184 (24.6%)	172 (31.7%)	1 (ref)	
	Divorced/ Separated	0 (0.0%)	41 (0.5%)	6 (1.1%)	1.85 (0.92 - 3.57)	
	Never married	2,444 (94.7%)	6,649 (74.9%)	365 (67.2%)	0.28 (0.25 - 0.31)	
***Occupation***	Farmer	71 (2.8%)	337 (3.8%)	35 (6.4%)	1 (ref)	
	Homemaker	47 (1.8%)	1,525 (17.2%)	90 (16.6%)	1.89 (1.45 - 2.46)	
	Labourer	288 (11.2%)	1,205 (13.6%)	175 (32.2%)	1.07 (0.82 - 1.39)	
	Service	129 (5.0%)	611 (6.9%)	44 (8.1%)	0.87 (0.65 - 1.16)	
	Student	1,986 (77.0%)	4,969 (56.0%)	185 (34.1%)	0.43 (0.34 - 0.54)	
	Unemployed	59 (2.3%)	227 (2.6%)	14 (2.6%)	0.69 (0.49 - 0.99)	
***Education***	No or primary schooling	1,398 (54.2%)	2,708 (30.5%)	135 (24.9%)	1 (ref)	1 (ref)
	Secondary	540 (21.0%)	2,081 (23.5%)	116 (21.4%)	1.94 (1.74 - 2.16)	**1.61** (1.41 - 1.82)*
	Higher Secondary	460 (17.9%)	2,914 (32.8%)	186 (34.3%)	2.95 (2.66 - 3.28)	**1.76** (1.54 - 2.01)*
	Graduate and above	179 (6.9%)	1,168 (13.2%)	106 (19.5%)	3.41 (2.95 - 3.96)	**1.81** (1.50 - 2.20)*
***Wealth index***	Lowest	275 (12.3%)	804 (11.3%)	72 (14.3%)	1 (ref)	1 (ref)
	Second	486 (21.8%)	1,351 (19.0%)	95 (18.8%)	0.90 (0.76 - 1.05)	0.92 (0.77 - 1.09)
	Middle	477 (21.4%)	1,536 (21.5%)	118 (23.4%)	1.04 (0.89 - 1.22)	0.95 (0.80 - 1.13)
	Fourth	478 (21.5%)	1,729 (24.3%)	113 (22.4%)	1.11 (0.94 - 1.30)	0.98 (0.83 - 1.17)
	Highest	512 (23.0%)	1,709 (24.0%)	107 (21.2%)	1.03 (0.88 - 1.20)	0.89 (0.75 - 1.07)
***Smokeless tobacco consumers***	No	2266 (87.8%)	7492 (84.4%)	399 (73.5%)	1 (ref)	1 (ref)
	Yes	314 (12.2%)	1,382 (15.6%)	144 (26.5%)	1.55 (1.38 - 1.75)	**1.53** (1.31 - 1.80)*
***Alcohol consumers (past 30 days)***	No	2565 (99.4%)	8689 (97.9%)	527 (97.1%)	1 (ref)	1 (ref)
	Yes	15 (0.6%)	185 (2.1%)	16 (2.9%)	2.52 (1.81 - 3.49)	**1.80** (1.19 - 2.72)*
***Salt consumption (grams per person per day)***	≤ 5 grams	121 (4.7%)	329 (3.7%)	36 (6.6%)	1 (ref)	
	> 5 grams	2,459 (95.3%)	8,545 (96.3%)	507 (93.4%)	1.05 (0.86 - 1.30)	
***Sugar consumption (grams per person per day)***	≤ 50 grams	2,032 (78.8%)	6,322 (71.2%)	355 (65.4%)	1 (ref)	1 (ref)
	> 50 grams	548 (21.2%)	2,552 (28.8%)	188 (34.6%)	1.50 (1.37 - 1.65)	**1.47** (1.32 - 1.63)*
***Fruit and vegetable servings per day***	≥ 5 servings per day	3 (0.1%)	3 (0.0%)	0 (0.0%)	1 (ref)	
	< 5 servings per day	2,571 (99.9%)	8,866 (100.0%)	543 (100.0%)	3.75 (0.73 - 20.1)	
***Adequately physically active***	No	1985 (76.9%)	5710 (64.3%)	410 (75.5%)	1 (ref)	
	Yes	595 (23.1%)	3,164 (35.7%)	133 (24.5%)	0.68 (0.62 - 0.74)	
***BMI categories***	Normal	1,586 (61.7%)	4,902 (56.4%)	289 (53.9%)	1 (ref)	1 (ref)
	Severe Underweight	205 (8.0%)	658 (7.6%)	34 (6.3%)	1.01 (0.86 - 1.18)	0.97 (0.81 - 1.16)
	Underweight	458 (17.8%)	1,726 (19.9%)	87 (16.2%)	1.15 (1.03 - 1.28)	1.03 (0.91 - 1.17)
	Overweight	179 (7.0%)	710 (8.2%)	63 (11.8%)	1.39 (1.19 - 1.63)	1.02 (0.85 - 1.21)
	Obesity	143 (5.6%)	695 (8.0%)	63 (11.8%)	1.65 (1.40 - 1.95)	0.99 (0.82 - 1.19)
***Hypertension***	Normal	1,495 (58.0%)	6,025 (67.9%)	1,495 (58.0%)	1 (ref)	
	Elevated	270 (10.5%)	722 (8.1%)	270 (10.5%)	0.70 (0.61 - 0.81)	
	HTN1	745 (28.9%)	1,949 (22.0%)	745 (28.9%)	0.84 (0.76 - 0.93)	
	HTN2	69 (2.7%)	177 (2.0%)	69 (2.7%)	0.98 (0.74 - 1.31)	
***Diabetes Mellitus status***	Normal	344 (93.5%)	1,073 (94.0%)	344 (93.5%)	1 (ref)	
	Pre-diabetic	19 (5.2%)	58 (5.1%)	19 (5.2%)	0.85 (0.53 - 1.38)	
	Diabetic	5 (1.4%)	10 (0.9%)	5 (1.4%)	0.70 (0.25 - 2.10)	
***Depression***	No	2490 (96.5%)	6011 (67.7%)	198 (36.5%)	1 (ref)	1 (ref)
	Yes	90 (3.5%)	2,863 (32.3%)	345 (63.5%)	8.41 (7.37 - 9.61)	**5.18** (4.41 - 6.10)*
***Anxiety***	No	2546 (98.7%)	7111 (80.1%)	423 (77.9%)	1 (ref)	1 (ref)
	Yes	34 (1.3%)	1,763 (19.9%)	120 (22.1%)	4.09 (3.60 - 4.66)	0.97 (0.82 - 1.15)
No. Obs.			9,711			
The model for stress shows odds adjusted for age category, sex, caste, education, wealth index, smokeless tobacco, alcohol in past 30 days, sugar consumption (grams per person per day), BMI categories, depression and anxiety.						

OR = Odds Ratio, aOR= adjusted Odds Ratio CI = Confidence Interval, OBC = Other backward class, SC = Scheduled caste, ST = Scheduled tribe, VJ/NT/Others = Vimukt jati/Nomadic Tribe/Others, BMI = Body Mass Index, HTN = Hypertension, TG = triglycerides, LDL = low density Lipoprotein, VLDL = Very low density Lipoprotein, HDL = high density Lipoprotein

*Indicates significant Odds ratios

As mentioned in Table 4, compared to the 10–19 years age group, those in the 20–30 age group have 1.82 times significant odds of reporting high perceived stress than reporting low or moderate stress combined. Males had significantly lower odds (aOR = 0.37) of high perceived stress than combined odds of feeling low or moderate stress when compared to females. Those from OBC caste had lower odds (aOR = 0.77) of perceiving high stress than moderate and low stress when compared to open caste. VJ/NT/Others caste when compared to open caste also had a significant protective role against high perceived stress than moderate and low stress combined. (aOR = 0.64). Those with higher levels of education when compared to those with no or primary education had higher odds (aOR of those secondary education = 1.61, aOR of those with higher secondary = 1.76, aOR of those with graduate degree or higher = 1.81) of perceiving high stress than feeling moderate and low stress. Current smokeless tobacco consumers had 1.53 times significant odds of perceiving higher stress than combined odds of low and moderate stress when compared to those who didn’t consume smokeless tobacco. Respondents who reported alcohol consumption in the past 30 days had 1.80 times more significant odds of experiencing high stress than combined perception of moderate and low stress than those who didn’t consume alcohol. Participants who consumed sugar more than 50 grams per day had 1.47 times odds of perceiving higher stress than combined odds of low and moderate stress when compared to those who consumed sugar within recommended limit. Participants with reported depression had 5.18 times significant odds of reporting higher stress than combined odds of low and moderate stress when compared to those who weren’t depressed.

Considering that the prevalence of consumption of smokeless tobacco was much higher compared to smoking in the age group 10–30 in our study area, we have presented the predictors of only smokeless tobacco consumption in Table 5. The variables considered in the best fitting model (Nagelkerke’s R² = 0.5358) are mentioned in the footnote of Table 5.

**Table 5:** Predictors for consumption of smokeless tobacco (Logistic regression model).

Characteristic (N)		Smokeless tobacco non- consumers	Smokeless tobacco consumers	OR (95% CI)	aOR (95% CI)
***Age categories (11,997)***	10-19	5,415 (53.3%)	210 (11.4%)	1 (ref)	1 (ref)
	20-30	4,742 (46.7%)	1,630 (88.6%)	8.86 (7.76 - 10.3)	**3.40** (2.60 - 4.46)*
***Sex (11,997)***	Female	5,741 (56.5%)	169 (9.2%)	1 (ref)	1 (ref)
	Male	4,416 (43.5%)	1,671 (90.8%)	12.9 (11.0 - 15.2)	**15.4** (11.4 - 20.9)*
***Caste (11,997)***	Open	441 (4.3%)	59 (3.2%)	1 (ref)	1 (ref)
	OBC	5,202 (51.2%)	733 (39.8%)	1.05 (0.80 - 1.41)	0.93 (0.62 - 1.42)
	SC	1,614 (15.9%)	235 (12.8%)	1.09 (0.81 - 1.49)	1.08 (0.69 - 1.70)
	ST	1,699 (16.7%)	543 (29.5%)	2.39 (1.80 - 3.22)	**1.78** (1.16 - 2.77)*
	VJ/NT/Others	1,201 (11.8%)	270 (14.7%)	1.68 (1.25 - 2.29)	1.42 (0.91 - 2.25)
***Marital Status (11,997)***	Currently married	2,014 (19.8%)	478 (26.0%)	1 (ref)	1 (ref)
	Divorced/ Separated	37 (0.4%)	10 (0.5%)	1.14 (0.53 - 2.22)	1.12 (0.35 - 3.07)
	Never married	8,106 (79.8%)	1,352 (73.5%)	0.70 (0.63 - 0.79)	**0.48** (0.38 - 0.61)*
***Occupation (11,997)***	Farmer	219 (2.2%)	224 (12.2%)	1 (ref)	1 (ref)
	Homemaker	1,589 (15.6%)	73 (4.0%)	0.04 (0.03 - 0.06)	**0.23** (0.15 - 0.37)*
	Labourer	710 (7.0%)	958 (52.1%)	1.32 (1.07 - 1.63)	1.09 (0.82 - 1.44)
	Service	524 (5.2%)	260 (14.1%)	0.49 (0.38 - 0.62)	**0.58** (0.43 - 0.80)*
	Student	6,871 (67.6%)	269 (14.6%)	0.04 (0.03 - 0.05)	**0.15** (0.11 - 0.20)*
	Unemployed	244 (2.4%)	56 (3.0%)	0.22 (0.16 - 0.31)	**0.49** (0.31 - 0.77)*
***Education (11,991)***	No or Primary	3,663 (36.1%)	578 (31.4%)	1 (ref)	1 (ref)
	Secondary	2,155 (21.2%)	582 (31.6%)	1.71 (1.51 - 1.94)	0.99 (0.80 - 1.22)
	Higher Secondary	2,983 (29.4%)	577 (31.4%)	1.23 (1.08 - 1.39)	**0.79** (0.64 - 0.97)*
	Graduate and above	1,350 (13.3%)	103 (5.6%)	0.48 (0.39 - 0.60)	**0.27** (0.19 - 0.37)*
***Wealth index (9,862)***	Lowest	876 (10.4%)	275 (19.1%)	1 (ref)	1 (ref)
	Second	1,604 (19.0%)	328 (22.8%)	0.65 (0.54 - 0.78)	**0.73** (0.57 - 0.94)*
	Middle	1,806 (21.4%)	325 (22.6%)	0.57 (0.48 - 0.69)	**0.57** (0.44 - 0.73)*
	Fourth	2,025 (24.0%)	295 (20.5%)	0.46 (0.39 - 0.56)	**0.62** (0.48 - 0.81)*
	Highest	2,110 (25.1%)	218 (15.1%)	0.33 (0.27- 0.40)	**0.47** (0.35 - 0.62)*
***Alcohol consumption in the last 30 days (11,997)***	No	10141 (99.8%)	1640 (89%)	1 (ref)	1 (ref)
	Yes	16 (0.2%)	200 (10.9%)	77.3 (47.9 - 134)	**16.5** (8.57 - 35.2)*
***Salt consumption (Grams per person per day) (11,997)***	≤ 5 grams	420 (4.1%)	66 (3.6%)	1 (ref)	1 (ref)
	> 5 grams	9,737 (95.9%)	1,774 (96.4%)	1.16 (0.90 - 1.52)	1.15 (0.79 - 1.70)
***Sugar consumption (Grams per person per day) (11,997)***	≤ 50 grams	7,542 (74.3%)	1,167 (63.4%)	1 (ref)	1 (ref)
	> 50 grams	2,615 (25.7%)	673 (36.6%)	1.66 (1.50 - 1.85)	**1.26** (1.07 - 1.48)*
***Fruit and vegetable servings per day (11,986)***	≥ 5 servings per day	5 (<0.1%)	1 (<0.1%)	1 (ref)	1 (ref)
	< 5 servings per day	10,143 (100.0%)	1,837 (99.9%)	0.91 (0.15 - 17.4)	0.50 (0.04 - 15.0)
***Adequate physical activity (11,997)***	Yes	2,896 (28.5%)	996 (54.1%)	1 (ref)	1 (ref)
	No	7261 (71.5%)	844 (45.9%)	0.34 (0.31 - 0.37)	**0.81** (0.69 - 0.95)*
***Stress (11,997)***	Low stress	2,266 (22.3%)	314 (17.1%)	1 (ref)	1 (ref)
	Moderate stress	7,492 (73.8%)	1,382 (75.1%)	1.33 (1.17 - 1.52)	**1.31** (1.08 - 1.59)*
	High perceived stress	399 (3.9%)	144 (7.8%)	2.60 (2.08 - 3.25)	0.98 (0.69 - 1.38)
***BMI (11,798)***	Normal	5,816 (58.4%)	961 (52.4%)	1 (ref)	1 (ref)
	Severe Underweight	750 (7.5%)	147 (8.0%)	1.19 (0.98 - 1.43)	1.33 (1.00 - 1.75)
	Underweight	1,857 (18.6%)	414 (22.6%)	1.35 (1.19 - 1.53)	1.17 (0.96 - 1.42)
	Overweight	806 (8.1%)	146 (8.0%)	1.10 (0.90 - 1.32)	**0.73** (0.56 - 0.96)*
	Obesity	736 (7.4%)	165 (9.0%)	1.36 (1.13 - 1.62)	0.89 (0.68 - 1.14)
***Hypertension (11,995)***	Normal	6,933 (68.3%)	861 (46.8%)	1 (ref)	1 (ref)
	Elevated	758 (7.5%)	267 (14.5%)	2.84 (2.42 - 3.31)	1.16 (0.91 - 1.48)
	HTN1	2,268 (22.3%)	634 (34.5%)	2.25 (2.01 - 2.52)	0.97 (0.81 - 1.16)
	HTN2	196 (1.9%)	78 (4.2%)	3.20 (2.43 - 4.19)	1.22 (0.81 - 1.83)
***Diabetes (1,599)***	Normal	1,274 (94.8%)	230 (90.2%)	1 (ref)	
	Pre-diabetic	57 (4.2%)	22 (8.6%)	2.14 (1.26 - 3.52)	
	Diabetic	13 (1.0%)	3 (1.2%)	1.28 (0.29 - 4.00)	
***Depression (11,997)***	No	7361 (72.5%)	1338 (72.7%)	1 (ref)	1 (ref)
	Yes	2,796 (27.5%)	502 (27.3%)	0.99 (0.88 - 1.10)	1.13 (0.94 - 1.36)
***Anxiety (11,997)***	No	8449 (83.2%)	1631 (88.6%)	1 (ref)	1 (ref)
	Yes	1,708 (16.8%)	209 (11.4%)	0.63 (0.54 - 0.74)	1.11 (0.86 - 1.43)
No. Obs: 9,704					
The model for smokeless tobacco consumption shows odds adjusted for age category, sex, caste, occupation, education, wealth index, alcohol in past 30 days, salt and sugar consumption (grams per person per day), fruit and vegetable consumption, adequate physical activity, stress, BMI categories, hypertension, depression and anxiety					

OR = Odds Ratio, aOR= adjusted Odds Ratio CI = Confidence Interval, OBC = Other backward class, SC = Scheduled caste, ST = Scheduled tribe, VJ/NT/Others = Vimukt jati/Nomadic Tribe/Others, BMI = Body Mass Index, HTN = Hypertension

*Indicates significant Odds ratios

Table 5 shows that compared to the 10–19 years age group, those in the 20–30 age group have 3.40 times significant odds of consuming smokeless tobacco. It was found that males have 15.4 times statistically significant odds of consuming smokeless tobacco than females. Respondents who identified as Scheduled Tribes have 1.78 times significant odds of consuming smokeless tobacco when compared to those from the Open category. Those respondents who were never married had significantly protective odds for consumption of smokeless tobacco (aOR = 0.48) than those who were married. Compared to farmers, the homemakers (aOR = 0.23, those in service (aOR = 0.58), students (aOR = 0.15) and those who were unemployed (aOR = 0.49) had significant odds of consuming smokeless tobacco and these associations were found to be statistically significant. Those who had education level of higher secondary (aOR = 0.79), graduation and above (aOR = 0.27) as well as respondents belonging to the second (aOR = 0.73), middle (aOR = 0.57), fourth (aOR = 0.62) and fifth quintile (aOR = 0.47) based on wealth index had significantly lower odds of consuming smokeless tobacco. Respondents who consumed alcohol in the past 30 days have significantly higher odds (aOR = 16.5) of consuming smokeless tobacco than those who didn’t consume alcohol. For every gram per person per day increase in sugar intake, the odds of consuming smokeless tobacco significantly increase by 1.26 times. Those not indulging in adequate physical activity had lower odds of consuming smokeless tobacco (aOR = 0.81). Those experiencing moderate stress had 1.31 times significant odds of consuming smokeless tobacco than those reporting low stress. Respondents who were overweight (aOR = 0.73) had lower odds for consumption of smokeless tobacco compared to those with normal BMI.

To study the predictors of physical inactivity a logistic regression model was constructed and the results are depicted in Table 6. The final best fit model has a Nagelkerke R^2^ value of 0.2079.

**Table 6:** Predictors for inadequate physical activity (logistic regression model).

Variables		Physically active	Physically inactive	OR (95% CI)	aOR (95% CI)
***Age categories (11,997)***	10-19	1,459 (37.5%)	4,166 (51.4%)	1 (ref)	1 (ref)
	20-30	2,433 (62.5%)	3,939 (48.6%)	0.57 (0.52 - 0.61)	**0.74** (0.64 - 0.85)*
***Sex (11,997)***	Female	1,168 (30.0%)	4,742 (58.5%)	1 (ref)	1 (ref)
	Male	2,724 (70.0%)	3,363 (41.5%)	0.30 (0.28 - 0.33)	**0.18** (0.16 - 0.21)*
***Caste (11,997)***	Open	183 (4.7%)	317 (3.9%)	1 (ref)	1 (ref)
	OBC	1,941 (49.9%)	3,994 (49.3%)	1.19 (0.98 - 1.43)	1.04 (0.82 - 1.33)
	SC	539 (13.8%)	1,310 (16.2%)	1.40 (1.14 - 1.73)	1.31 (1.00 - 1.72)
	ST	733 (18.8%)	1,509 (18.6%)	1.19 (0.97 - 1.45)	1.16 (0.89 - 1.51)
	VJ/NT/Others	496 (12.7%)	975 (12.0%)	1.13 (0.92 - 1.40)	1.26 (0.95 - 1.66)
***Marital status (11,997)***	Currently married	927 (23.8%)	1,565 (19.3%)	1 (ref)	1 (ref)
	Divorced/Separated	17 (0.4%)	30 (0.4%)	1.05 (0.58 - 1.95)	1.17 (0.53 - 2.83)
	Never married	2,948 (75.7%)	6,510 (80.3%)	1.31 (1.19 - 1.43)	**1.96** (1.69 - 2.28)*
***Occupation (11,997)***	Farmer	288 (7.4%)	155 (1.9%)	1 (ref)	
	Homemaker	604 (15.5%)	1,058 (13.1%)	3.25 (2.62 - 4.06)	
	Labourer	814 (20.9%)	854 (10.5%)	1.95 (1.57 - 2.43)	
	Service	300 (7.7%)	484 (6.0%)	3.00 (2.36 - 3.83)	
	Student	1,826 (46.9%)	5,314 (65.6%)	5.41 (4.42 - 6.63)	
	Unemployed	60 (1.5%)	240 (3.0%)	7.43 (5.30 - 10.5)	
***Education (11,991)***	No or Primary	1,210 (31.1%)	3,031 (37.4%)	1 (ref)	1 (ref)
	Secondary	1,057 (27.2%)	1,680 (20.7%)	0.63 (0.57 - 0.70)	**0.72** (0.63 - 0.83)*
	Higher Secondary	1,158 (29.8%)	2,402 (29.6%)	0.83 (0.75 - 0.91)	0.98 (0.86 - 1.13)
	Graduate and above	464 (11.9%)	989 (12.2%)	0.85 (0.75 - 0.97)	0.96 (0.79 - 1.16)
***Wealth index (9,862)***	Lowest	311 (11.0%)	840 (11.9%)	1 (ref)	1 (ref)
	Second	571 (20.2%)	1,361 (19.4%)	0.88 (0.75 - 1.04)	0.88 (0.74 - 1.06)
	Middle	611 (21.6%)	1,520 (21.6%)	0.92 (0.78 - 1.08)	0.98 (0.82 - 1.17)
	Fourth	640 (22.6%)	1,680 (23.9%)	0.97 (0.83 - 1.14)	1.04 (0.87 - 1.25)
	Highest	696 (24.6%)	1,632 (23.2%)	0.87 (0.74 - 1.02)	0.94 (0.78 - 1.13)
***Smokeless tobacco consumption (11,997)***	No	2896 (74.4%)	7261 (89.6%)	1 (ref)	1 (ref)
	Yes	996 (25.6%)	844 (10.4%)	0.34 (0.31 - 0.37)	**0.79** (0.69 - 0.92)*
***Alcohol consumed in last 30 days (11,997)***	No	3743 (96.2%)	8038 (99.2%)	1 (ref)	1 (ref)
	Yes	149 (3.8%)	67 (0.8%)	0.21 (0.16 - 0.28)	**0.51** (0.35 - 0.73)*
***Salt consumption (Grams per person per day) (11,997)***	≤ 5 grams	117 (3.0%)	369 (4.6%)	1 (ref)	1 (ref)
	> 5 grams	3,775 (97.0%)	7,736 (95.4%)	0.65 (0.52 - 0.80)	**0.72** (0.55 - 0.93)*
***Sugar consumption (Grams per person per day) (11,997)***	≤ 50 grams	2,642 (67.9%)	6,067 (74.9%)	1 (ref)	1 (ref)
	> 50 grams	1,250 (32.1%)	2,038 (25.1%)	0.71 (0.65 - 0.77)	**0.87** (0.79 - 0.97)*
***Fruit and Vegetable servings per day (11,986)***	≥ 5 servings per day	1 (0.0%)	5 (0.1%)	1 (ref)	1 (ref)
	< 5 servings per day	3,889 (100.0%)	8,091 (99.9%)	0.42 (0.02 - 2.58)	0.44 (0.02 - 2.95)
***Stress (11,997)***	Low stress	595 (15.3%)	1,985 (24.5%)	1 (ref)	1 (ref)
	Moderate stress	3,164 (81.3%)	5,710 (70.5%)	0.54 (0.49 - 0.60)	**0.44** (0.38 - 0.50)*
	High perceived stress	133 (3.4%)	410 (5.1%)	0.92 (0.75 - 1.15)	0.84 (0.65 - 1.09)
***BMI*** ***(11,798)***	Normal	2,135 (55.7%)	4,642 (58.3%)	1 (ref)	1 (ref)
	Sev Underweight	282 (7.4%)	615 (7.7%)	1.00 (0.86 - 1.17)	**1.32** (1.09 - 1.60)*
	Underweight	759 (19.8%)	1,512 (19.0%)	0.92 (0.83 - 1.01)	1.11 (0.98 - 1.27)
	Overweight	308 (8.0%)	644 (8.1%)	0.96 (0.83 - 1.11)	1.04 (0.88 - 1.25)
	Obesity	351 (9.2%)	550 (6.9%)	0.72 (0.62 - 0.83)	**0.82** (0.69 - 0.99)*
***Hypertension (11,995)***	Normal	2,517 (64.7%)	5,277 (65.1%)	1 (ref)	1 (ref)
	Elevated	403 (10.4%)	622 (7.7%)	0.74 (0.64 - 0.84)	1.18 (1.00 - 1.39)
	HTN1	878 (22.6%)	2,024 (25.0%)	1.10 (1.00 - 1.21)	**1.80** (1.59 - 2.04)*
	HTN2	94 (2.4%)	180 (2.2%)	0.91 (0.71 - 1.18)	**1.57** (1.16 - 2.14)*
***Diabetes (1,599)***	Normal	517 (95.0%)	987 (93.6%)	1 (ref)	
	Pre-diabetic	21 (3.9%)	58 (5.5%)	1.45 (0.88 - 2.46)	
	Diabetic	6 (1.1%)	10 (0.9%)	0.87 (0.32 - 2.58)	
***Depression (11,997)***	No	2829 (72.7%)	5870 (72.4%)	1 (ref)	1 (ref)
	Yes	1,063 (27.3%)	2,235 (27.6%)	1.01 (0.93 - 1.10)	0.94 (0.82 - 1.07)
***Anxiety (11,997)***	No	3385 (87.0%)	6695 (82.6%)	1 (ref)	1 (ref)
	Yes	507 (13.0%)	1,410 (17.4%)	1.41 (1.26 - 1.57)	**1.51** (1.28 - 1.79)*
No. Obs.: 9,704					
The model for inadequate physical activity shows odds adjusted for age category, sex, caste, education, wealth index, smokeless tobacco consumption, alcohol in past 30 days, salt and sugar consumption (grams per person per day), fruit and vegetable consumption, stress, BMI categories, hypertension, depression and anxiety					

OR = Odds Ratio, aOR= adjusted Odds Ratio CI = Confidence Interval, OBC = Other backward class, SC = Scheduled caste, ST = Scheduled tribe, VJ/NT/Others = Vimukt jati/Nomadic Tribe/Others, BMI = Body Mass Index, HTN = Hypertension

*Indicates significant Odds ratios

As seen in Table 6, respondents in the age group 20–30 years had significantly lower odds of being physically inactive (aOR = 0.74). It was found that males have 0.18 times significant odds of not indulging in physical activity compared to females. Respondents who were never married had 1.96 times more significant odds of not being physically active than those who were married at the time of data collection. Those who had completed their secondary schooling had 0.72 times significant odds of being inadequately physically active compared to those who had no or primary schooling. Those who reported consuming smokeless tobacco had 0.79 times significant odds and those who consumed alcohol in last 30 days had 0.51 times significant odds of not being physically active than those who didn’t consume it. Those consuming excess salt (> 5 grams) and excess sugar (>50 grams) had 0.72 and 0.87 times significant odds of not indulging in adequate physical activity. Participants who reported moderate stress had 0.44 times significant odds of being inadequately physically active compared to those with low stress. Respondents who were severely underweight and obese had 1.32 and 0.82 times significant odds of being physically inactive than those with normal BMI. Participants classified as HTN grade I and II were found to have significantly higher odds of being physically inactive (aOR= 1.80 and 1.57 respectively). Those respondents who reported feeling anxious have 1.51 times significant odds to be physically inactive. (Table 6)

To understand the predictors of excess salt (Nagelkerke’s R^2^ = 0.131) and excess sugar consumption (Nagelkerke’s R^2^ = 0.118), we developed a logistic regression model. This model created for understanding predictors for excess salt consumption included age categories, sex, caste, wealth index, smokeless tobacco, alcohol and sugar consumption, physical activity, stress, hypertension, depression, anxiety, BMI categories and diabetes status. Among the demographic variables, those from the second wealth quintile have 3.15 times the odds to have excess salt consumption compared to those who belong to the lowest wealth quintile. Respondents reporting excess sugar consumption have significantly higher odds (aOR = 5.05) of consuming an excess amount of salt. Those who reported symptoms of depression had significantly lower odds of consuming excess salt (aOR = 0.48) than those who reported no depression. Respondents who had elevated blood pressure and those categorised as having pre-diabetes had significantly lower odds of consuming excess amounts of salt (aOR = 0.38 and 0.30 respectively) of consuming an excess salt. (Table 7)

**Table 7:** Predictors of excess salt and sugar consumption (Logistic regression).

Variables		Excess salt consumption				Excess sugar consumption			
		Adequate	Excess	OR (95% CI)	aOR (95% CI)	Adequate	Excess	OR (95% CI)	aOR (95% CI)
***Age categories (11,997)***	10-19	216 (44.4%)	5,409 (47.0%)	1 (ref)	1 (ref)	4,269 (49.0%)	1,356 (41.2%)	1 (ref)	1 (ref)
	20-30	270 (55.6%)	6,102 (53.0%)	0.90 (0.75 - 1.08)	1.19 (0.66 – 2.17)	4,440 (51.0%)	1,932 (58.8%)	1.37 (1.26 - 1.49)	0.79 (0.57 - 1.11)
***Sex (11,997)***	Female	264 (54.3%)	5,646 (49.0%)	1 (ref)	1 (ref)	4,637 (53.2%)	1,273 (38.7%)	1 (ref)	1 (ref)
	Male	222 (45.7%)	5,865 (51.0%)	1.24 (1.03 - 1.48)	1.03 (0.55 - 1.93)	4,072 (46.8%)	2,015 (61.3%)	1.80 (1.66 - 1.96)	**1.63 (1.19 - 2.23)**
***Caste (11,997)***	Open	34 (7.0%)	466 (4.0%)	1 (ref)	1 (ref)	354 (4.1%)	146 (4.4%)	1 (ref)	1 (ref)
	OBC	233 (47.9%)	5,702 (49.5%)	1.79 (1.21 - 2.55)	0.75 (0.12 – 2.75)	4,109 (47.2%)	1,826 (55.5%)	1.08 (0.88 - 1.32)	0.77 (0.43 - 1.39)
	SC	58 (11.9%)	1,791 (15.6%)	2.25 (1.44 - 3.46)	1.01 (0.14 - 4.52)	1,446 (16.6%)	403 (12.3%)	0.68 (0.54 - 0.85)	**0.33 (0.17 - 0.66)**
	ST	88 (18.1%)	2,154 (18.7%)	1.79 (1.17 - 2.66)	0.63 (0.09 – 2.66)	1,712 (19.7%)	530 (16.1%)	0.75 (0.61 - 0.93)	**0.39 (0.20 - 0.76)**
	VJ/NT/Others	73 (15.0%)	1,398 (12.1%)	1.40 (0.91 - 2.11)	0.56 (0.08 - 2.43)	1,088 (12.5%)	383 (11.6%)	0.85 (0.68 - 1.07)	0.50 (0.25 - 1.02)
***Marital status (11,997)***	Currently married	155 (31.9%)	2,337 (20.3%)	1 (ref)		1,935 (22.2%)	557 (16.9%)	1 (ref)	
	Divorced/Separated	1 (0.2%)	46 (0.4%)	3.05 (0.66 - 54.2)		37 (0.4%)	10 (0.3%)	0.94 (0.44 - 1.83)	
	Never married	330 (67.9%)	9,128 (79.3%)	1.83 (1.50 - 2.23)		6,737 (77.4%)	2,721 (82.8%)	1.40 (1.27 - 1.56)	
***Occupation (11,997)***	Farmer	16 (3.3%)	427 (3.7%)	1 (ref)		245 (2.8%)	198 (6.0%)	1 (ref)	
	Homemaker	102 (21.0%)	1,560 (13.6%)	0.57 (0.32 - 0.95)		1,303 (15.0%)	359 (10.9%)	0.34 (0.27 - 0.43)	
	Laborer	67 (13.8%)	1,601 (13.9%)	0.90 (0.50 - 1.52)		1,131 (13.0%)	537 (16.3%)	0.59 (0.47 - 0.73)	
	Service	36 (7.4%)	748 (6.5%)	0.78 (0.42 - 1.40)		498 (5.7%)	286 (8.7%)	0.71 (0.56 - 0.90)	
	Student	257 (52.9%)	6,883 (59.8%)	1.00 (0.58 - 1.62)		5,316 (61.0%)	1,824 (55.5%)	0.42 (0.35 - 0.52)	
	Unemployed	8 (1.6%)	292 (2.5%)	1.37 (0.59 - 3.41)		245 (2.8%)	198 (6.0%)	0.48 (0.35 - 0.66)	
***Education (11,991)***	No or Primary	183 (37.7%)	4,058 (35.3%)	1 (ref)		3,215 (36.9%)	1,026 (31.2%)	1 (ref)	1 (ref)
	Secondary	97 (20.0%)	2,640 (22.9%)	1.23 (0.96 - 1.58)		2,043 (23.5%)	694 (21.1%)	1.06 (0.95 - 1.19)	0.76 (0.53 - 1.09)
	Higher Secondary	148 (30.5%)	3,412 (29.7%)	1.04 (0.83 - 1.30)		2,481 (28.5%)	1,079 (32.8%)	1.36 (1.23 - 1.51)	1.17 (0.82 - 1.69)
	Graduate and above	58 (11.9%)	1,395 (12.1%)	1.08 (0.81 - 1.48)		966 (11.1%)	487 (14.8%)	1.58 (1.39 - 1.80)	1.57 (0.94 - 2.62)
***Wealth index (9,862)***	Lowest	44 (10.5%)	1,107 (11.7%)	1 (ref)	1 (ref)	865 (12.2%)	286 (10.3%)	1 (ref)	1 (ref)
	Second	63 (15.0%)	1,869 (19.8%)	1.18 (0.79 - 1.74)	**3.15 (1.07 - 9.93)**	1,445 (20.4%)	487 (17.5%)	1.02 (0.86 - 1.21)	0.88 (0.53 - 1.45)
	Middle	99 (23.6%)	2,032 (21.5%)	0.82 (0.56 - 1.16)	1.42 (0.54 - 3.5)	1,514 (21.4%)	617 (22.1%)	1.23 (1.05 - 1.45)	0.76 (0.46 - 1.25)
	Fourth	80 (19.0%)	2,240 (23.7%)	1.11 (0.76 - 1.61)	1.31 (0.50 - 3.22)	1,640 (23.2%)	680 (24.4%)	1.25 (1.07 - 1.47)	0.82 (0.51 - 1.35)
	Highest	134 (31.9%)	2,194 (23.2%)	0.65 (0.45 - 0.91)	0.88 (0.34 - 2.18)	1,612 (22.8%)	716 (25.7%)	1.34 (1.15 - 1.58)	0.73 (0.44 - 1.23)
***Smokeless tobacco consumption (11,997)***	No	420 (86.4%)	9737 (84.6%)	1 (ref)	1 (ref)	7542	2615	1 (ref)	1 (ref)
	Yes	66 (13.6%)	1,774 (15.4%)	1.16 (0.90 - 1.52)	1.94 (0.79 - 5.35)	1,167 (13.4%)	673 (20.5%)	1.66 (1.50 - 1.85)	1.17 (0.77 - 1.76)
***Alcohol consumed in last 30 days (11,997)***	No	481 (99.0%)	11300 (98.2%)	1 (ref)	1 (ref)	8577	3204	1 (ref)	1 (ref)
	Yes	5 (1.0%)	211 (1.8%)	1.80 (0.82 - 5.07)	0.75 (0.12 – 14.9)	132 (1.5%)	84 (2.6%)	1.70 (1.29 - 2.24)	1.98 (0.74 - 5.24)
***Salt consumption (11,997)***	≤ 5 grams	NA				440 (5.1%)	46 (1.4%)	1 (ref)	1 (ref)
	> 5 grams					8,269 (94.9%)	3,242 (98.6%)	3.75 (2.79 - 5.16)	**5.25 (2.36 – 14.0)**
***Sugar consumption (11,997)***	≤ 50 grams	440 (90.5%)	8,269 (71.8%)	1 (ref)	1 (ref)	NA			
	> 50 grams	46 (9.5%)	3,242 (28.2%)	3.75 (2.79 - 5.16)	**5.05 (2.27 - 13.5)**				
***Fruit and vegetable servings per day (11,986)***	≥ 5 servings per day	0 (0.0%)	6 (0.1%)			3 (0.0%)	3 (0.1%)	1 (ref)	
	< 5 servings per day	484 (100.0%)	11,496 (99.9%)			8,698 (100.0%)	3,282 (99.9%)	0.38 (0.07 - 2.04)	
***Adequate physical activity (11,997)***	No	369 (75.9%)	7736 (67.2%)	1 (ref)	1 (ref)	6067	2038	1 (ref)	1 (ref)
	Yes	117 (24.1%)	3,775 (32.8%)	0.65 (0.52 - 0.80)	1.19 (0.64 - 2.14)	2,642 (30.3%)	1,250 (38.0%)	0.71 (0.65 - 0.77)	0.79 (0.59 - 1.06)
***Stress (11,997)***	Low stress	121 (24.9%)	2,459 (21.4%)	1 (ref)	1 (ref)	2,032 (23.3%)	548 (16.7%)	1 (ref)	1 (ref)
	Moderate stress	329 (67.7%)	8,545 (74.2%)	1.28 (1.03 - 1.58)	1.86 (1.00 - 3.46)	6,322 (72.6%)	2,552 (77.6%)	1.50 (1.35 - 1.66)	**1.44 (1.03 - 2.03)**
	High perceived stress	36 (7.4%)	507 (4.4%)	0.69 (0.48 - 1.03)	0.62 (0.24 - 1.74)	355 (4.1%)	188 (5.7%)	1.96 (1.61 - 2.40)	**1.91 (1.07 - 3.41)**
***Hypertension (11,995)***	Normal	257 (52.9%)	7,537 (65.5%)	1 (ref)	1 (ref)	5,739 (65.9%)	2,055 (62.5%)	1 (ref)	1 (ref)
	Elevated	39 (8.0%)	986 (8.6%)	0.86 (0.62 - 1.23)	**0.38 (0.17 - 0.90)**	681 (7.8%)	344 (10.5%)	1.41 (1.23 - 1.62)	0.96 (0.60 - 1.51)
	HTN1	183 (37.7%)	2,719 (23.6%)	0.51 (0.42 - 0.62)	0.56 (0.30 - 1.05)	2,094 (24.0%)	808 (24.6%)	1.08 (0.98 - 1.19)	1.21 (0.88 - 1.65)
	HTN2	7 (1.4%)	267 (2.3%)	1.30 (0.66 - 3.07)	0.64 (0.16 - 4.34)	193 (2.2%)	81 (2.5%)	1.17 (0.90 - 1.52)	0.97 (0.43 - 2.03)
***Depression (11,997)***	No	296 (60.9%)	8403 (73.0%)	1 (ref)	1 (ref)	6379	2320	1 (ref)	1 (ref)
	Yes	190 (39.1%)	3,108 (27.0%)	0.58 (0.48 - 0.70)	**0.48 (0.25 – 0.96)**	2,330 (26.8%)	968 (29.4%)	1.14 (1.05 - 1.25)	**2.02 (1.44 - 2.83)**
***Anxiety (11,997)***	No	354 (72.8%)	9726 (84.5%)	1 (ref)	1 (ref)	7218	2862	1 (ref)	1 (ref)
	Yes	132 (27.2%)	1,785 (15.5%)	0.49 (0.40 - 0.61)	1.62 (0.67 - 4.42)	1,491 (17.1%)	426 (13.0%)	0.72 (0.64 - 0.81)	0.97 (0.64 - 1.47)
***BMI categories (11,798)***	Normal	269 (56.8%)	6,508 (57.5%)	1 (ref)	1 (ref)	4,940 (57.7%)	1,837 (56.7%)	1 (ref)	1 (ref)
	Sev Underweight	40 (8.4%)	857 (7.6%)	0.89 (0.64 - 1.26)	1.82 (0.52 – 11.5)	675 (7.9%)	222 (6.8%)	0.88 (0.75 - 1.04)	1.26 (0.75 - 2.06)
	Underweight	95 (20.0%)	2,176 (19.2%)	0.95 (0.75 - 1.21)	1.00 (0.53 - 1.99)	1,646 (19.2%)	625 (19.3%)	1.02 (0.92 - 1.14)	0.85 (0.60 - 1.19)
	Overweight	33 (7.0%)	919 (8.1%)	1.15 (0.81 - 1.69)	1.86 (0.68 – 6.59)	665 (7.8%)	287 (8.9%)	1.16 (1.00 - 1.34)	1.21 (0.76 – 1.90)
	Obesity	37 (7.8%)	864 (7.6%)	0.97 (0.69 - 1.39)	1.52 (0.61 - 4.41)	631 (7.4%)	270 (8.3%)	1.15 (0.99 - 1.34)	1.05 (0.67 - 1.65)
***Diabetes (1,599)***	Normal	64 (85.3%)	1,440 (94.5%)	1 (ref)	1 (ref)	1,086 (94.4%)	418 (93.1%)	1 (ref)	1 (ref)
	Pre-diabetic	9 (12.0%)	70 (4.6%)	0.35 (0.17 - 0.77)	**0.30 (0.13 - 0.71)**	51 (4.4%)	28 (6.2%)	1.43 (0.88 - 2.27)	1.54 (0.90 - 2.61)
	Diabetic	2 (2.7%)	14 (0.9%)	0.31 (0.08 - 2.01)	0.57 (0.09 - 11.1)	13 (1.1%)	3 (0.7%)	0.60 (0.14 - 1.87)	0.50 (0.10 - 1.78)
No. Obs.		1,291				1,291			
		The model for salt shows odds adjusted for age category, sex, caste, wealth index, smokeless tobacco consumption, alcohol in past 30 days, sugar consumption (grams per person per day), stress, BMI categories, hypertension, diabetes, depression and anxiety				The model for sugar shows odds adjusted for age category, sex, caste, wealth index, smokeless tobacco consumption, alcohol in past 30 days, sugar consumption (grams per person per day), fruit and vegetable consumption, stress, BMI categories, hypertension, diabetes, depression and anxiety			

OR = Odds Ratio, aOR= adjusted Odds Ratio CI = Confidence Interval, OBC = Other backward class, SC = Scheduled caste, ST = Scheduled tribe, VJ/NT/Others = Vimukt jati/Nomadic Tribe/Others, BMI = Body Mass Index, HTN = Hypertension,

*Indicates significant Odds ratios.

A best fitting model was created for understanding predictors for sugar consumption which included age categories, sex, caste, education, wealth index, smokeless tobacco, alcohol and salt consumption, physical activity, stress, hypertension, depression, anxiety, BMI categories and diabetes. Males had around 63% significantly higher odds of having excess sugar consumption compared to females. Those from SC and ST caste had significantly lower odds of 0.33 and 0.39 respectively of consuming excess amounts of sugar than those coming from Open caste. For those who consumed excess salt per person per day, the odds of consuming excess sugar increased significantly by 5.25 times. Respondents who reported moderate and high levels of stress had 1.44 and 1.91 times higher odds respectively of consuming sugar in excess amounts than those who had low stress whereas those who reported depression had 2.02 times the odds for the same. (Table 7)

## Discussion

This paper discusses results of the baseline cross-sectional survey of the V-CaN trial. It reports the estimates of behavioral and biochemical risk factors for various NCDs. This study also suggests possible predictors of these behavioral risk factors in the age group of 10–30 years old individuals. The characteristics such as occupation, education and marital status were non-comparable mainly due to age categories, i.e., 10–19 and 20–30 years. The study identified the prevalence of some common behavioral and biochemical risk factors in the study area, which includes overall prevalence of less than recommended consumption of fruits and vegetables (99.9%), excess salt intake (95.9%), excess sugar intake (27.4%), smokeless tobacco users (15.3%) and smokers (0.3%), alcohol consumers (1.8%), inadequate physical activity (32.4%), overweight and obesity (8.1% and 7.6% respectively), moderate (74%) and high (4.5%) perceived stress, anxiety (16%) and depression (27.5%).

In the study, it was observed that the average number of fruits and vegetable servings per day is far less (1.5 servings) than the expected minimum servings, i.e., 5 servings for leading a healthy life as per WHO guidelines of healthy diet [16,25,26]. Possible reasons for low consumption of fruits and vegetables can be their availability in the rural areas, affordability of fruits, cultural dietary patterns or preference for packaged or junk food [27].

The study showed that the age group 20–30 years had significantly higher risk of developing stress as compared to 10–19 years age group, whereas a study conducted in Gujarat, India on youth showed there is no significant relationship between age groups and stress in youth [28]. Male participants were having significantly lesser odds for developing stress as compared to female youth participants in the study which was also supported by a study conducted in Madhya Pradesh, India [29]. The odds of developing stress among OBC and Vimukt jati (VJ) was lower as compared to other castes. Though we did not find any article talking about the stress in VJ category people as compared to other castes, we did find articles about higher stress among scheduled caste people and OBC as compared to other higher Hindu castes which was opposite to what current study showed regarding OBCs [30,31]. The study shows the association between education and stress in youth. As compared to no or primary education, secondary or higher education levels have higher chances of developing stress, which was also shown in various studies conducted in India [28,32,33]. The study also suggests that smokeless tobacco (SLT) consumers had higher odds of developing stress, which was aligned with another study conducted in India [34]. The study also suggested significantly higher odds of stress in participants with depression as compared with those who were not having depression which was also aligned with findings of a study conducted with school going adolescents in India [35]. The study also shows that there are significantly higher odds of stress in alcohol consumers, which are in sync with the findings of the study conducted in India which talks about the reciprocal relationship between alcohol consumption and stress [36]. The higher prevalence of stress in this age group can be attributed to multiple reasons like changes in their body due to puberty, demands of school like performing well, peer pressure or issues with friends, financial problems in the family, unsafe environment at home or school, pressure to get a job/source of income, dysfunctional family dynamics, etc. [37]

The average prevalence of smokeless tobacco (SLT) users was high (15.3%) as compared to smokers (0.3%). The prevalence of smokeless tobacco users was higher in the 20–30 age group, i.e., 26.9% in rural areas which was also found in the Global Adult Tobacco Survey-2 published in 2020 [38]. This shows the need for early intervention in young age groups for better health outcomes in later life. The study shows a higher number of SLT consumers were males as compared to females. Similar results were also seen in the study from 2023 using data from Longitudinal Ageing Study in India (LASI) wave 1 and a report on tobacco control in India 2022 [39,40]. Among students aged 13–15, 8.4% acknowledged currently using tobacco, as per the Global Youth Tobacco Survey 2 (GYTS), whereas The Global Adult Tobacco Survey-2 (GATS-2) found that tobacco use was more common as people aged, and this was true for both smoking and SLT items that contained tobacco, which aligns with the findings of the current study [40]. The study shows higher odds of SLT consumption in ST category as compared with other caste categories, which also aligns with the results from the analysis of Global Adult Tobacco Survey (GATS) 2016–17 [41]. The occupation and its type showed a significant role in increased consumption of SLT. The unemployed, homemakers, students and service people have significantly lower odds of SLT consumption as compared to laborious work doing individuals. This finding also aligns with the findings from the LASI data study [39]. Additionally, compared to those with less education than graduation, the study indicates that those who have graduated and above had decreased odds of consuming SLT, which aligns with the findings from the LASI study [39]. However, as this study involves teenagers who were yet to complete their education, a large number of them might still be enrolled in school. Thus, it’s possible that the prevalence was under-reported due to social desirability bias. The study shows significant inverse association between the SLT consumption and wealth index of the population which also coincides with the study conducted in India [42,43]. The number of alcohol consumers using SLT was also more as compared to non-alcohol consumers, which also aligns with the findings from a study on National Sample Survey [44] while another study suggests the existence of spatial heterogeneity across different states and districts of India [45]. The study shows higher odds for consuming SLT with higher sugar consumption per day as well as moderate stress levels which is in accordance with other study suggesting a strong association between these variables [37]. The current study also suggests the significant inverse association with the adequate physical activity and SLT consumption which also aligns with the findings from a study conducted in India [43]. The odds of having lower BMI (Severe underweight) are high and getting overweight are low in people consuming smokeless tobacco as compared to people with normal BMI which has also shown from the results of various studies conducted in India [46,47].

The average prevalence of adequately physically active young individuals from both the age categories was very less, i.e., approximately one third of the total, which was in sync with the studies conducted in different parts of the world [48,49]. The study shows statistically significant differences in physical activity status based on age category and sex. The study shows the level of inadequate physical activity decreases with increase in age group, while a study in Brazil shows that in adults, as the age increases the level of physical activity decreases [50]. Males were seen to be more physically active than females, which also aligns with findings from different studies conducted in India [49,51,52]. The study shows that the single people are having higher odds in engaging in inadequate physical activity as compared with the married and divorced participants, which contradicts the findings of a study conducted in Brazil [50]. The study suggests the participants with secondary education tend to have lower odds of inadequate physical activity as compared to other levels of education, which contradicts the findings from a study conducted in Spain [53]. A study also highlights the inverse relationship between physical activity and SLT consumption, which coincides with the findings from the study conducted in India [43]. This is most likely due to the fact that those who are more physically active tend to be more health-conscious. It was found that alcohol consumers are less likely to have inadequate physical activity as compared to those without alcohol consumption, which coincides with the findings from a Brazilian study [53]. The study shows that adolescents with moderate levels of stress are more predisposed to indulge in physical activity. Physical activity was found to be more inadequate in those having anxiety, whereas the study conducted with a group of female students in 2020 showed no effect of high physical activity on stress levels but aligned with these study results regarding the correlation between physical activity and anxiety [54]. The study suggests higher odds of having hypertension with higher inadequate physical activity, which aligns with the findings from a review [55]. The study shows higher odds of having inadequate physical activity with severely underweight people and lower odds of inadequate physical activity with obese people, which is contradicting with the findings from the study conducted in the UK [56]. Even though the study shows that those who consume more salt and sugar are having a negative likelihood of inadequate physical activity, any direct association of the same was not found. However, the strong link between dietary patterns and physical activity can’t be disregarded.

The average sugar consumption was found to be 44.7 grams per person per day which was less than the recommended limits by WHO, i.e., 50 grams per day, but for additional health benefits, the sugar intake should be less than 5% of the total which is less than 25 grams per person per day [18], which indicates space for improvement in the daily eating habits. The study shows statistically significant differences in sugar consumption in case of sex, i.e., Males have significantly higher odds of consuming appropriate amounts of sugar as compared to females, but a study conducted in a city of Karnataka state with school going adolescents showed no significant difference based on sex [57]. The difference in the two studies might be due to the study setting being different, i.e., rural vs urban. Mean (SD) for salt intake was 8.7 (3.1) grams per person per day in both the groups which is higher than the recommended salt intake by WHO [17] and it was comparable with the results from a multicentric study conducted in 2017 in India [58]. The study signifies association of salt consumption with wealth index as the second quintile of the wealth index has significantly higher odds of appropriate salt intake as compared with lowest, while a study conducted on south Indian population showed higher the income, higher would be the salt consumption [59]. The study showed elevated blood pressure (BP) significantly decreases the odds of having appropriate salt consumption as compared to normal BP. The participants with moderate stress level were having higher chances of consuming appropriate amounts of salt as compared to low stress people, but the review published in 2010 showed that acute stress has no effect on salt consumption, however, chronic stress may have effect on salt consumption [60]. The study shows higher odds of consuming excess sugar in participants with depression as compared with those not having depression, which aligns with the results from a systematic review and meta-analysis conducted in China [61]. The SC/ST caste showed lesser odds for the consumption of appropriate amounts of sugar. We were unable to find this relation between these two variables in the literature. The study shows higher odds for consuming appropriate amounts of salt in high SLT consuming people, but we could not find any previous study which can show relation between the two variables.

A large sample was surveyed through simple random sampling which reduces potential risk of selection bias. Our study has used the WHO STEPS questionnaire which is a standardized tool which makes the results of this study comparable with other international studies. Thus, the data analysis was performed as per the standard cutoffs and guidelines available for analysis like the analysis guide was used for analyzing data on physical activity. Very few such studies are available from India which have been conducted on such a large sample in this age group. We weren’t able to find Indian reference values for biochemical tests in adolescent age groups and thus have used American cut-offs for a few variables. The cut-offs suggested by our study can thus be tested on a larger sample size for correctness and standardization for Indian use. Since the behavioral risk factors like dietary habits of consuming salt, sugar, fruits and vegetables;

addictions like tobacco and alcohol consumption; physical activity were self-reported by the respondents, social desirability bias cannot be ruled out specially in this age group. We however tried to conduct the interviews in a confidential and non-judgmental manner to minimize any such bias affecting the study results. Since, the respondents were from the rural area, generalizability of these findings in the urban area is limited.

Thus, this study informs about predictors of some common modifiable risk factors of NCDs. Along with other lifestyle changes suggested as preventive measures for delaying onset of NCDs, interventions should also focus on supporting the mental health of individuals. This can be achieved by stronger integration of the National Mental Health Program with the National Program for Non communicable diseases (NP-NCD) and other National programs related to health of adolescents. Community based assessment checklist (CBAC) is currently being used by frontline workers under the NP-NCD program for community based risk assessment of individuals above 30 years of age. Based on the findings of this study, we would recommend that this risk factor assessment should be initiated for everyone beyond 10 years of age. The screening and risk profiling for NCDs starts after 30 years of age, however this paper brings to light that risk profiling in 10–30 years of age is equally important as these are important habit forming years and interventions targeted to reduce risk factors in this age might help to reduce the overall prevalence of NCDs or atleast delay their onset. A brief summary of what this study adds is mentioned in Table 8.

**Table 8:** What this study adds.

Sr. No.	What is known	This study adds to the existing body of knowledge
**1.**	Common risk factors for NCDs	Predictors for risk factors of NCDs
**2.**	Lifestyle habits develop during the early years of life and influenced during adolescence	Strength of association between the various risk factors during this age
**3.**	Prevalence of NCDs in adults and little literature around prevalence of risk factors and NCDs in adolescents	Prevalence of risk factors as well as hypertension and diabetes in this age group on such a large sample
**4.**		Our study includes not only the behavioural risk factors but also the biochemical risk factors in a sub group of 1600 study participants.

## Conclusion

The prevalence of risk factors for NCDs is notably high among adolescents, underscoring the importance of frequent screening through comprehensive surveys. Such proactive measures can enable timely interventions aimed at reducing the incidence of NCDs. While current policies predominantly address tobacco consumption through smoking regulations, there is a pressing need for more stringent laws concerning the use of chewable tobacco. Strengthening the screening of adolescents for various NCDs and their associated risk factors into existing programs, such as the Rashtriya Kishor Swasthya Karyakram, Rashtriya Bal Swasthya Karyakram, National Programme for Non-communicable diseases, to ensure that these issues are acknowledged by the public health system. By doing so, we can take timely, policy-based actions to address and mitigate the impact of NCDs among the youth.

### Trial Registration details

Clinical Trials Registration India CTRI/2020/10/028700; https://ctri.nic.in/Clinicaltrials/showallp.php?mid1=47597&EncHid=&userName=V-CaN

International Registered Report Identifier (IRRID): DERR1-10.2196/42450

## Supporting information

S1 Fig Figure suggesting simplified flow of selection of participants in the baseline assessment of the trial. (TIF)

S1 Checklist STROBE checklist. (DOCX)

S1 Protocol VCaN study protocol. (PDF)
