# Supplementary material for: Prevalence and predictors of risk factors for non-communicable diseases in individuals aged 10–30 years: Findings from baseline cross-sectional assessment of V-CaN
Source: PLOS Glob Public Health. 2026 Oct 30;6(10):e0005956. doi: 10.1371/journal.pgph.0005956 (PMC13722311; doi:10.1371/journal.pgph.0005956)
Supplement: Supplementary file 2 [file pgph.0005956.s002.docx]

STROBE Statement—Checklist of items that should be included in reports of ***cross-sectional studies***

|  | Item No | Recommendation | Page No |
| --- | --- | --- | --- |
| **Title and abstract** | 1 | (*a*) Indicate the study’s design with a commonly used term in the title or the abstract | Title page |
|  |  | (*b*) Provide in the abstract an informative and balanced summary of what was done and what was found | Page 1-2 |
| Introduction | | | |
| Background/rationale | 2 | Explain the scientific background and rationale for the investigation being reported | Page 3-4, line 28-61 |
| Objectives | 3 | State specific objectives, including any prespecified hypotheses | Page 4, line 62-65 |
| Methods | | | |
| Study design | 4 | Present key elements of study design early in the paper | Page 4, line 69-70; page 5, line 78-85 |
| Setting | 5 | Describe the setting, locations, and relevant dates, including periods of recruitment, exposure, follow-up, and data collection | Page 4, line 70; Page 5, line 75-85 |
| Participants | 6 | (*a*) Give the eligibility criteria, and the sources and methods of selection of participants | Page 5, line 80-87 |
| Variables | 7 | Clearly define all outcomes, exposures, predictors, potential confounders, and effect modifiers. Give diagnostic criteria, if applicable | Page 5, line 86-87; the details for the same are mentioned in the Trial protocol attached as a supplementary file |
| Data sources/ measurement | 8* | For each variable of interest, give sources of data and details of methods of assessment (measurement). Describe comparability of assessment methods if there is more than one group | Page 5-7, line 91-131 |
| Bias | 9 | Describe any efforts to address potential sources of bias | Page 5, line 88-90 |
| Study size | 10 | Explain how the study size was arrived at | Page 5, line 86-88; trial protocol attached as a supplementary file |
| Quantitative variables | 11 | Explain how quantitative variables were handled in the analyses. If applicable, describe which groupings were chosen and why | Page 6, line 96-100 |
| Statistical methods | 12 | (*a*) Describe all statistical methods, including those used to control for confounding | Page 6-7, line 97-125 |
|  |  | (*b*) Describe any methods used to examine subgroups and interactions | Page 7, line 128-134 |
|  |  | (*c*) Explain how missing data were addressed | Page 7, line 125-127 |
|  |  | (*d*) If applicable, describe analytical methods taking account of sampling strategy |  |
|  |  | (*e*) Describe any sensitivity analyses |  |
| Results | | | |
| Participants | 13* | (a) Report numbers of individuals at each stage of study—eg numbers potentially eligible, examined for eligibility, confirmed eligible, included in the study, completing follow-up, and analysed | Figure 1 |
|  |  | (b) Give reasons for non-participation at each stage | Figure 1 |
|  |  | (c) Consider use of a flow diagram | Figure 1 |
| Descriptive data | 14* | (a) Give characteristics of study participants (eg demographic, clinical, social) and information on exposures and potential confounders | Page 8-10, Table 1 |
|  |  | (b) Indicate number of participants with missing data for each variable of interest | Page 8-10, Table 1 |
| Outcome data | 15* | Report numbers of outcome events or summary measures | Page 11-14, Table 2a and Table 2b |
| Main results | 16 | (*a*) Give unadjusted estimates and, if applicable, confounder-adjusted estimates and their precision (eg, 95% confidence interval). Make clear which confounders were adjusted for and why they were included | Page 15-24, Table 3; Page 25-32, Table 4; Page 34-40, Table 5; Page 42-52, Table 6 |
|  |  | (*b*) Report category boundaries when continuous variables were categorized | Page 6-7, line 101-125 |
|  |  | (*c*) If relevant, consider translating estimates of relative risk into absolute risk for a meaningful time period |  |
| Other analyses | 17 | Report other analyses done—eg analyses of subgroups and interactions, and sensitivity analyses |  |
| Discussion | | | |
| Key results | 18 | Summarise key results with reference to study objectives | Page 53, line 298-304 |
| Limitations | 19 | Discuss limitations of the study, taking into account sources of potential bias or imprecision. Discuss both direction and magnitude of any potential bias | Page 58-59, line 423-426 |
| Interpretation | 20 | Give a cautious overall interpretation of results considering objectives, limitations, multiplicity of analyses, results from similar studies, and other relevant evidence | Page 59, table of interpretation- This study adds to the existing body of knowledge |
| Generalisability | 21 | Discuss the generalisability (external validity) of the study results | Page 58-59, line 418-419, 425-426 |
| Other information | | | |
| Funding | 22 | Give the source of funding and the role of the funders for the present study and, if applicable, for the original study on which the present article is based | Page 60, line 451-454 |

*Give information separately for exposed and unexposed groups.

**Note:** An Explanation and Elaboration article discusses each checklist item and gives methodological background and published examples of transparent reporting. The STROBE checklist is best used in conjunction with this article (freely available on the Web sites of PLoS Medicine at http://www.plosmedicine.org/, Annals of Internal Medicine at http://www.annals.org/, and Epidemiology at http://www.epidem.com/). Information on the STROBE Initiative is available at www.strobe-statement.org.
