# Supplementary material for: Prevalence and predictors of risk factors for non-communicable diseases in individuals aged 10–30 years: Findings from baseline cross-sectional assessment of V-CaN
Source: PLOS Glob Public Health. 2026 Oct 30;6(10):e0005956. doi: 10.1371/journal.pgph.0005956 (PMC13722311; doi:10.1371/journal.pgph.0005956)
Supplement: Supplementary file 4 [file pgph.0005956.s005.docx]

Dear Editors and reviewers,

Thank you for taking out time and reviewing the manuscript and the detailed feedback. We appreciate the efforts from your side and have tried to address as many responses as we can and have modified the manuscript in accordance with the comments received. We are hopeful that with these edits, the manuscript will have a better clarity and be easy to understand. Please find our responses to each of the points raised in the following table**:**

| **Comments** | **Authors’ response** | **Line and page number** |
| --- | --- | --- |
| 1. In the ethics statement in the Methods, you have specified that verbal consent was obtained. Please provide additional details regarding how this consent was documented and witnessed, and state whether this was approved by the IRB | Thank you for the comment.  The participants were asked for consent verbally by the data enumerator and documented as a part of the questionnaire. The questionnaire tool was designed in a way to end the form if the consent was denied by the respondent. | Page 7, line 90-94 |
| 2. Please provide separate figure files in .tif or .eps format. | Thank you for the comment.  We have changed the file format to suit the requirements of the journal and reuploaded. | Online submission form |
| 3. We have noticed that you have uploaded Supporting Information files, but you have not included a list of legends. Please add a full list of legends for your Supporting Information files after the references list. | Thank you for bringing this out. We have now added a list of Supporting Information Files after the references in the manuscript. | Page 54, line 706-714 |
| 4. In the online submission form, you indicated that “The data that support the findings of this study are available from the corresponding author upon reasonable request.”.  All PLOS journals now require all data underlying the findings described in their manuscript to be freely available to other researchers, either  1. In a public repository,  2. Within the manuscript itself, or  3. Uploaded as supplementary information. | Based on the requirements of the journal, we have now modified our data availability statement to include the link to an online public repository. The data file can be accessed using the following link: <https://doi.org/10.5281/zenodo.19368119> | The data availability statement was modified in the online submission form. |

**Additional editor comments**

| **Comments** | **Authors’ response** | **Line and page number** |
| --- | --- | --- |
| 1. The Introduction is well-written in terms of presenting the burden of NCDs and citing national datasets such as NFHS and CNNS. However, it may be helpful to strengthen the rationale slightly to more clearly justify the need for conducting this large baseline assessment. | Thank you for the comment.  The larger study (V-CaN project) was a hybrid implementation trial aimed at studying the implementation as well as effectiveness of the interventions. To estimate the effectiveness, we estimated a sample size of 12000 and 1600 individuals for behavioural and biochemical risk factors respectively. In this paper, we present the findings from this first cross sectional survey (baseline survey) under this study. We have added the sample size estimation and this justification line in the methods section as without the sample size estimation, it would have become difficult for readers to relate if the text was added in the introduction section. | Page 6, lines 66-70 |
| 2. At present, the paragraphs primarily describe existing prevalence data. It would add clarity if a few sentences explicitly explained how this study differs from or adds to existing datasets, particularly given that CNNS and other national surveys have already reported adolescent risk factors. Clarifying what specific gap this study addresses (for example, extension of the age range to 30 years, district-level HDSS-based profiling, integration of behavioral and biochemical measures, or linkage to the implementation arm of the V-CaN trial) would strengthen the justification. | Thank you for the comment.  We have added this as a part of our introduction. | Page 6-7, lines 73--79 |
| 3. Introduce your study clearly and boldly, early introduce your protocol paper and then it may be useful to state clearly that this manuscript presents baseline findings intended to inform and tailor the implementation component of the hybrid trial. Making this linkage more explicit would help readers better appreciate the added value and purpose of the baseline survey. Otherwise, readers may be high with the thought that this study would give us something from A hybrid type 2 implementation effectiveness trial, whereas, in current form it is just a cross-sectional study. | Thank you for highlighting this.  We have introduced this as a part of introduction. | Page 6-7, lines 71-79 |
| In Methods |  |  |
| 1. I would suggest clearly adding standard subheadings for easy reading. | Thank you for the comment.  Subheadings like study setting, study population, sample size estimation, data collection and analysis and operational definitions have been added as a part of the Materials and Methods section. | Page 7-11 |
| 2. Given the number of variables assessed in this study, it may be helpful to consider presenting a consolidated table in the Methods section summarizing the operational definitions and measurement approaches used. This could include definitions of outcome variables, biochemical assessment methods (including laboratory techniques and cut-offs), procedures for estimating salt intake in grams per person per day, and the method used to assess fruit and vegetable servings and physical activity. | Thank you for the suggestion.  We had earlier included these details in a descriptive manner, we have now added it as a table under Methodology (Sub-section of “Operational definitions”). | Page 9-11, Table 1 |
| 3. 10-20 and 20-30 years: rationale for these categories. consider aligning with The World Health Organization (WHO) or similar body, that provides specific definitions for different age groups within the young population. According to WHO, adolescents are defined as individuals between 10 and 19 years of age, young people encompass those in the age group of 10-24, and youth are categorized as those aged 15-24. Currently we are overlapping. | Thank you for the comment. The age groups were 10-19 and 20-30. We have modified this in the analysis as well as in the manuscript. | Page 11-38 |
| Results |  |  |
| 1. Table 3: Association of stress with socio-demographic and other behavioral characteristics among 10-30-year-old persons. Why are we presenting this as a separate table, given that it was less discussed while building the rationale? Need more clarity on this. I would look for more bar charts comparing prevalence between male, female, urban/rural, and so on in a baseline paper. | Thank you for the comment. Stress was one of the behavioral risk factors studied. Stress was assessed using perceived stress score. As reported in Table 3a, 74% respondents have reported a moderate amount of stress which prompted us to consider studying which factors affect the degree of stress perceived by the respondents.  As there are many variables which were considered in this baseline assessment, the bar charts are looking clustered. However if the reviewers or Editor insists, we are open to plot them.  The prevalence of risk factors as per each variable under study has currently been reported in Tables 4 to 7 under the Results section. | Page 17, line 191 |
| 2. table 4: only talks about Smokeless tobacco. rationale? We can consider tobacco as a unit, if the sample for smokers was less. is it going to change our recommendations anyways? | Based on the prevalence reported in Table 3a, smokeless tobacco was much more prevalent in this age group. Considering this, we wanted to study the factors affecting the indulgence in smokeless tobacco consumption in this age group. Hence we have reported prevalence of smoking as well as smokeless tobacco use, however while analysing, we have considered creating a model only for smokeless tobacco use. | Page 22, line 222-224 |
| 3. while doing regression, please recheck multicollinearity, as currently there are so many variables that may explain causal pathways. example: role of marital status while doing regression for salt and sugar. | Thank you for the comment. We have already looked for multicolinearity and variables showing moderate to high collinearity have been omitted from the analysis.  This has been mentioned in the methods section. | Page 11, line 132-139 |
| 4. Tables in general are very cluttered, which affects readability and makes inference. | We have edited the tables for better readability. | Page 11 - 38 |
| Discussion |  |  |
| 1. Please add 4 key messages from your study, that would help u during the intervention or would inform policy, and some recommendations at the end of the discussion. | Thank you for the comment.  We have modified our discussion based on your suggestions. | Page 45, line 451- 462 |

**Reviewer 1 comments**

| Comments | Authors’ response | Line and page number |
| --- | --- | --- |
| - please use the background, methods, results, conclusions subheadings. | Thank you for the comment.  We have revised our headings following the journal guidelines and put the heads as Introduction, Materials and methods, Results, Discussion, Conclusion. | Throughout the manuscript. |
| Indicate more clearly that this is not the results of a trial, but an examination of baseline data among trial participants for a different purpose. | Thank you for the comment.  We have modified the introduction part to bring this early and more clearly in the manuscript. | Page 6-7, lines 71-79 |
| It is unclear whether there are 12,000 participants who provided data, or if only 1,600 provided data for analysis? How many patients in the V-CaN hybrid type 2 implementation effectiveness trial? | Thank you for the comment.  The survey intended to capture 2 types of risk factors - i) behavioural, ii) biochemical. For the behavioural risk factors the sample size was 12000. From amongst these 12000 participants those who consented for giving the blood samples, 1600 respondents were selected using simple random sampling for assessing the prevalence and determinants of biochemical risk factors.  We have edited figure 1 with simplified participant flow. | Figure 1 uploaded as a supplementary file |
| What is the main aim of this trial and when will that be reported? | Thank you for the comment.  The main aim of this trial is to assess the implementation outcomes and effectiveness of community based strategies for primary prevention of NCDs. The trial has 4 arms, with 3 arms representing different change agents (Women’s SHG, School students, VHNSCs,) and the fourth arm being the control arm. The findings of the trial are currently being analysed and will be submitted for publication in the second half of 2026. We have added a few lines in introduction for better clarity around the overall objectives of the trial and introduced our protocol paper in the introduction part so that readers can refer to the trial protocol for further details. | Page 6, Line 71-72 |
| "We have also created a best-fit regression model for risk factors as outcome variables" What are the outcome variables? predictors? Please lead the reader through to understand why this was done. | We have added more detailed statement to simplify these terms for the reader. | Page 3, line 12-14 |
| - it is not clear what these abbreviations signify: RKSK, RBSK, NP-NCD, NFHS. | Thank you for the comment.  We have removed the abbreviations from the abstract as well as the main manuscript. We have retained only the full forms in the later part of discussion | Page 4, line 28; Page 47, line 473-474 |
| -was the prevalence of smoking only 0.3%? | Thank you for the comment.  Yes, the reported prevalence of smoking was 0.3% only. Since the survey was conducted in youths, social desirability bias cannot be ruled out. We have stated this as a limitation. | Page 45, line 447 |
| line 34: don't use DM without explaining it. | Thank you for the comment.  We have stated the full form of DM and HTN while introducing the abbreviations. | Page 5, line 38-39 |
| line 35 HTN? is this the same definition of high blood pressure mentioned in line 39? If so, use same terms. | Thank you for the comment.  We have modified the term to HTN | Page 5, line 43 |
| Why higher percentages of high blood pressure in 10-12 year olds compared to 13-19 year olds? | Thank you for the comment.  We would like to clarify here that this is not a finding from our study but from the analysis of CNNS conducted during 2016-2018 in India, which has been included in the introduction to highlight that NCDs are increasing in India in younger age groups. The authors from that article have mentioned a few reasons for high prevalence of increased BP, but have not discussed the reasons for the difference in blood pressure across the two age groups. Since this is not from our study, it would be difficult for us to comment on the reasons for the same. | - |
| line 93: was the data cleaned using R studio? How? | Thank you for the comment.  We have added the names of packages used for the cleaning process under the heading of “Materials and Methodology”, sub heading of “Data collection and analysis”. | Page 8-9, line 118-119 |
| line 109: (perhaps give context to 600 met-minutes - i.e. ~ an hour of running, for example, if appropriate. | Thank you for the comment.  This is a good suggestion for explaining the interpretations of our study, however, since at this point we are objectively describing the cutoffs that we have used for reporting our results, subjective examples might not be appropriate at this point and hence we are refraining from such contextualization. | - |
| line 120: Capitals for American Academy of Pediatrics. | Thank you for the comment.  This has been included in a table (Table 1) of operational definitions. We have incorporated the suggestions there. | Page 10 |
| Table 1: is Other backward class the best descriptor? | Thank you for the comment.  Yes, it is a standard caste category used by the Government of India. The **Other Backward Class** (**OBC**) is a collective term used by the [Government of India](https://en.wikipedia.org/wiki/Government_of_India) to classify [communities](https://en.wikipedia.org/wiki/Caste) that are "educationally or socially backward" (i.e., deprived/ disadvantaged). It is one of several official classifications of the population of India, along with [general castes](https://en.wikipedia.org/wiki/Forward_caste), [Scheduled Castes and Scheduled Tribes](https://en.wikipedia.org/wiki/Scheduled_Castes_and_Scheduled_Tribes) (SCs and STs). | - |
| Could there be a figure to show the predictors of each outcome? | Thank you for the comment.  This could have been done. However, the tables of predictors with the OR and aOR are self explanatory. Making a figure for the same would lead to duplication of information and hence we avoided it. In case the reviewers feel that this is necessary, we are still open to adding the figure. | - |
| line 227: should be 0.50 | Thank you for the comment.  We have made the suggested change | Page 26, Line 236 |
| line 227: compared to a farmer and laborer | Thank you for the comment.  In this analysis, farmers were the reference group hence the statement was correct. We have not described laborers as they did not show any significant association for tobacco consumption. | - |
| Instead of gender, should it be sex? | Thank you for the comment.  We have changed gender to sex at all places for uniformity and appropriateness as per your suggestions. | Throughout the manuscript. |
| Were the blood samples only tested for blood glucose status? | Thank you for the comment.  No, the blood samples were tested for Lipid panel in addition to blood glucose. The same has already been described in table 3b. | Page 14, line 160; Page 17, Table 3b |

**Reviewer 2 comments**

| Comments | Authors’ response | Line and page number |
| --- | --- | --- |
| 1. Clarification on participant flow  Please clarify what happened to participants who consented for testing but were not included.  You mention that ~11,000 agreed, but the final sample was 1,600.  A clear flow diagram (screened → consented → included → analyzed) with reasons for exclusion would improve transparency and reduce potential selection bias concerns. | Thank you for the comment.  We had to take only 1600 blood samples and 9850 had consented for blood sample testing. We did not take blood samples for around 8250 individuals. Since the blood sample was drawn immediately after the survey for behavioral risk factors was completed, only the selected 1600 respondents were contacted. For the others, we analysed only the information pertaining to behavioral risk factor survey.  Based on your suggestion, we have edited the figure 1 with details of participation flow. | Figure 1 submitted as supporting file. |
| 2. Regression modelling approach  In the prediction models, consider including only biologically plausible predictors for each outcome.  For example, for stress, variables such as psychosocial factors may be more appropriate.  For salt intake, predictors like dietary patterns or processed food consumption would be more relevant.  Some associations (e.g., smoking with higher salt intake, or stress with vegetable intake) may not be conceptually necessary and could introduce overadjustment or model instability. | Thank you for your comment.  We have attempted to report associations between the risk factors. Since, these risk factors frequently co-exist with each other, we wanted to study the presence of which risk factors significantly affects behaviour of common risk factors such as physical inactivity, smokeless tobacco consumption, stress, excess salt and sugar consumption.  Models have been created only after checking for multi-collinearity between all variables being considered. Those with moderate or high collinearity have been eliminated. We have attempted to create best fit models considering model coefficients like R squared value, AIC and BIC values. Some of these models have low explanatory potential and the same has been reported as values of model coefficients in the results section.  We have revised our regression tables based on your suggestions. | - |
| 3. Variable selection strategy  Instead of stepwise methods, an enter (forced entry) method based on conceptual framework is preferable.  This helps avoid data-driven selection and preserves meaningful associations grounded in theory. | Thank you for this valuable input.  We attempted the forced entry model for all outcome variables based on the conceptual framework published in the protocol paper cited in the manuscript.  Most of the variables excluded from our models earlier as well were solely removed due to multicollinearity between the variables. Hence, there wasn’t much difference between the odds that were reported and the ones that are being reported now. Still we have now reported the newer models in our results section. | Page 11, line 132-139; Page 17- 37. |
| 4. Interpretation of findings  The discussion should expand on the biological and behavioral plausibility of key associations.  For example, explain why salt intake might relate to physical activity (e.g., dietary patterns, sports drinks, higher caloric intake).  Similar explanatory reasoning with supporting literature should be provided for other significant findings. | Thank you for the comment.  We have modified our discussion based on your suggestions. | Page 38-45; lines 318-320; 338-344; 356-358; 365; 373; 394; 408; 417; 445-462 |
| 5. Strengthening the discussion  Link findings more explicitly to existing evidence and mechanisms, not just statistical significance.  Highlight how results inform adolescent NCD prevention strategies or policy implications. | Thank you for the comment.  We have modified our discussion based on your suggestions. | Page 44-46 |
