# Supplementary material for: Prevalence and predictors of risk factors for non-communicable diseases in individuals aged 10–30 years: Findings from baseline cross-sectional assessment of V-CaN
Source: PLOS Glob Public Health. 2026 Oct 30;6(10):e0005956. doi: 10.1371/journal.pgph.0005956 (PMC13722311; doi:10.1371/journal.pgph.0005956)
Supplement: Supplementary file 5 [file pgph.0005956.s004.docx]

**Response to Reviewer’s comments**

| **Sr No.** | **Query** | **Response** |
| --- | --- | --- |
| 1 | Please ensure that the Author List in your manuscript file matches the Author List in the online submission form, including initials and order. Currently, author "Mudita Joshii" name listed in manuscript file is different from online submission form.  Please use either initials or full names in both the manuscript and online submission form. | Thank you for pointing that out. We have corrected the author name in the Title Page of the manuscript under the Authors and affiliations section. |
| 2 | If you are submitting a clinical trial, it must be registered in a recognized international registry, e.g. ClinicalTrials.gov or the International Standard Randomized Controlled Trial Number (ISRCTN) Registry.  Please include the name of the registry, the trial number, and the trial URL in the Methods section of your manuscript file. | This was previously included as a separate heading “Trial Registration details” immediately before the References. We have now included these as a part of Methodology on page 4-5, line 70-73. |
| 3 | Clinical Trials Registration Number (usually NCT or ISRCTN number) present in the Abstract section of the Manuscript file; | We have added the requested details in the abstract on page 1, lines 5-8. |
| 4 | A CONSORT 2025 Checklist or relevant reporting guideline (uploaded as a Supporting Information file) - a copy of the up-to-date checklist version may be accessed at: https://www.consort-spirit.org/ | CONSORT checklist has been prepared and is being attached as a supporting information file. However, we are reporting findings from the baseline survey of the trial and hence we are also uploading STROBE checklist for the present article as a supporting information file. |
| 5 | CONSORT Flow Diagram (uploaded as Fig 1); | We have revised our Figure 1 as per the template available. It has information relevant only to the recruitment of participants as we are currently reporting only baseline survey findings. |
| 6 | A Trial Protocol - please provide the most recently approved version, including any amendments, with the date of the change noted (uploaded as a Supporting Information file); | We have referenced the Trial protocol as reference number 11 in the manuscript. We are also uploading the trial protocol as a supporting information file. |
| 7 | Details of prior approval for human subjects research by an institutional review board (IRB) or equivalent ethics committee(s) in the Methods section, including board or committee names and approval numbers. | We have added these details on Page 5, line 75-78 in the methodology section. |
| 8 | We also recommend that authors of clinical trial studies provide a SPIRIT 2025 checklist. You can read more about the 2025 SPIRIT updates and access a copy of the checklist at:  https://www.consort-spirit.org/ | Since we aren’t reporting the trial protocol, we are not currently submitting SPIRIT checklist. However, if the reviewers still insist that we submit it, we are open to submitting it. |
| 9 | Please complete all items on the Human Participants Research Checklist that are relevant for your submission, by following this link: http://journals.plos.org/plosone/s/file? id=dc11/PLOSOne_Human_Subjects_Research_Checklist.docx. There may be overlap between the checklist items and other queries listed here; please address any duplicated queries both in your response email and on the checklist itself. Upload the completed Human Participants Research checklist as file type "Other" when you re-submit your manuscript. This document is for internal journal use only and will not be published if your article is accepted. The requested information will help us to assess whether your submission complies with PLOS's policies and adheres to applicable reporting standards. Note that your manuscript may be rejected if you provide incomplete or inadequate responses to the checklist questions and that changing the 'Section/Category' of your article does not affect this requirement. | We are uploading the Human Participants Research Checklist. |
| 10 | Your study includes live participants, but you have not included an Ethics Statement. Please include an Ethics Statement subsection to your Materials and Methods, to include:  i) The name(s) of the Institutional Review Boards) or Ethics Committee(s)  ii) The approval number(s), or a statement that approval was granted by the named board (s)  iii) (for human participants or donors) - A statement that formal consent was obtained (must state whether verbal/written) OR the reason consent was not obtained (e.g., anonymity). | We have included Ethics statement as a part of Methodology, Page 5, line 75-78. Along with these, for details of the name of Institutional board and members of the board, we are uploading the Ethics approval letter as a supplementary file. |
| 11 | Adding exact dates of recruitment period in methodology section | As suggested, we have included the dates on page 5, line 76. |
