# Supplementary material for: Prevalence and predictors of risk factors for non-communicable diseases in individuals aged 10–30 years: Findings from baseline cross-sectional assessment of V-CaN
Source: PLOS Glob Public Health. 2026 Oct 30;6(10):e0005956. doi: 10.1371/journal.pgph.0005956 (PMC13722311; doi:10.1371/journal.pgph.0005956)
Supplement: Supplementary file 6 [file pgph.0005956.s006.docx]

Dear Editors and reviewers,

Thank you for taking out time and reviewing the manuscript and the detailed feedback. We appreciate your efforts and have tried to address the queries to the best of our ability and have modified the manuscript in accordance with the comments received. We are hopeful that with these edits, the manuscript will have a better clarity and be easy to understand. Please find our responses to each of the points raised in the following table**:**

**Reviewer #3:** Kalantri et al. submitted a manuscript titled “Prevalence and predictors of risk factors for non-communicable diseases in individuals aged 10–30 years: Findings from baseline assessment of V CaN – A hybrid type 2 implementation effectiveness trial.” While the research topic is important and has the potential to contribute meaningfully to the literature, the manuscript has substantial methodological and scientific shortcomings. Issues span from the description of the sample size calculation to the analytical approach, and the overall reporting lacks scientific rigor. In addition, several sections are unclear, inconsistent, or do not adhere to established reporting standards such as STROBE.

| **Comments** | **Authors’ response** | **Line and page number** |
| --- | --- | --- |
| 1) Title, study design, and objectives: The manuscript presents conflicting descriptions of the study design and objectives. The title refers to an “implementation effectiveness trial,” the introduction describes the work as “implementation research,” the methods section labels it a “cross-sectional survey,” and the authors’ response to previous reviewer comments states that the analysis is based on baseline data from trial participants. These inconsistencies undermine the clarity and coherence of the study. Similar inconsistencies appear throughout the manuscript and make the study not scientifically sound. | Thank you for the comment.  We have made necessary changes for the text to be more coherent throughout the manuscript. | Throughout the manuscript |
| 2) Sample size calculation: The stated aim is to estimate prevalence; however, the sample size calculation does not follow standard methods for prevalence estimation. For prevalence studies, the recommended approach is the formula z^2 pq/d^2, which appropriately accounts for the expected prevalence and desired precision. The current method is not aligned with accepted epidemiologic practice. | Thank you for the comment.  We understand the way of articulating the statement may have led to confusion.  We clarify that this was not a separate prevalence study. The sample size was calculated to assess the effectiveness of the trial interventions and all the selected respondents have undergone the baseline assessment. The assumptions for assessing the effectiveness have already been stated in the manuscript. We have modified the section of sample size estimation and now explicitly mention that the actual sample size was calculated to assess the effectiveness of the V-CaN trial. We have also added “The baseline assessment for behavioral and biochemical risk factors was carried out in all the 12,000 and 1600 respondents respectively. In the present manuscript, we present the findings of this baseline assessment.” | Page 8, line 127-135 |
| 3) Analytical approach: The regression analyses appear to be entirely data driven, with no underlying causal framework or theoretical justification guiding variable selection. In epidemiologic research, risk factor analyses require a clear causal rationale to avoid biased or uninterpretable estimates. Without incorporating subject matter knowledge or causal structure (e.g., directed acyclic graphs, conceptual models), the current models are scientifically inappropriate and may yield misleading results. | Thank you for the comment.  We agree with the point that a causal framework/ biological plausibility must guide variable selection while making regression models. We have also made a few changes to our model based on your comment.  Although we admit that we did not construct DAGs for each model, we also reassure that the variable selection was based on both biological plausibility as well as results of bivariate analysis. The investigators had a detailed discussion on which variables to include in the regression models.  Most of the variables selected in the models have some epidemiological evidence of associations. However, we also included a few variables which might not have a clear established association, but were significantly/ near significantly associated on bivariate analysis. These were included to address any confounding/ effect modification that might have resulted due to them.  Further, a few variables although being statistically associated on bivariate analysis as well as having a clear biological plausibility had to be excluded since they were demonstrating multicollinearity (e.g. physical activity in the model of stress, Diabetes in the model of tobacco consumption, diabetes and occupation in the model of physical activity and a few more) or were distorting the results due to less number of events and hence imprecise results (e.g. fruits and veg in stress) . Since there were many models that we are presenting here, we thought it would have been difficult for the readers to imbibe all these findings amidst the large number of associations and models that we are presenting, and hence we decided against presenting these details in the manuscript.  You would also notice that there a few variables that have been excluded as they did not satisfy any of the statistical or biological plausibility criteria (e.g. salt consumption/fruits and veg with stress, marital status in salt and sugar consumption) | Page 12, line 170-172;  Page 35 line 313-325;  Page 41, line 330-340 |
| 4) Overall writing: The overall writing quality is insufficient for publication. Several sections are unclear, contain errors, or lack the precision expected in scientific manuscripts. | Thank you for the comment. We have tried to modify the language to improve clarity throughout the manuscript. | Throughout the manuscript |

**Reviewer #4:**

| **Comments** | **Author s’ response** | **Line and page number** |
| --- | --- | --- |
| I would like to thank the authors for the opportunity to review this manuscript. The study addresses an important and relevant topic and has the potential to contribute meaningfully to the existing literature.  The manuscript is generally well organized, and the objectives are clearly stated. The methodology appears appropriate for the research question, and the results are presented in a logical manner. However, several areas would benefit from further clarification and revision to improve the overall quality and readability of the manuscript. I did not identify any major concerns regarding research ethics or publication ethics based on the information provided. Subject to satisfactory revision of the points noted above, the manuscript may be suitable for publication. | Thank you for the comment. We have tried to modify the language to improve clarity throughout the manuscript. | Throughout the manuscript |
