# Supplementary material for: Prevalence and predictors of risk factors for non-communicable diseases in individuals aged 10–30 years: Findings from baseline cross-sectional assessment of V-CaN
Source: PLOS Glob Public Health. 2026 Oct 30;6(10):e0005956. doi: 10.1371/journal.pgph.0005956 (PMC13722311; doi:10.1371/journal.pgph.0005956)
Supplement: Peer review history

# Author comment: Author response to Decision Letter 0

**DOI:** 10.1371/journal.pgph.0005956.r001

16 Jan 2026

Attachment Submitted filename: Response to Reviewer_PLOS.docx

# Peer review: Decision Letter 0

**DOI:** 10.1371/journal.pgph.0005956.r002

**Authors:** Madhur Verma

22 Feb 2026

PGPH-D-25-03509

Prevalence and predictors of risk factors for non-communicable diseases in individuals aged 10-30 years: Findings from baseline assessment of V-CaN – A hybrid type 2 implementation effectiveness trial

PLOS Global Public Health

Dear Dr. Raut,

Thank you for submitting your manuscript to PLOS Global Public Health. After careful consideration, we feel that it has merit but does not fully meet PLOS Global Public Health’s publication criteria as it currently stands. Therefore, we invite you to submit a revised version of the manuscript that addresses the points raised during the review process.

Please submit your revised manuscript by Apr 08 2026 11:59PM. If you will need more time than this to complete your revisions, please reply to this message or contact the journal office at globalpubhealth@plos.org. When you're ready to submit your revision, log on to https://www.editorialmanager.com/pgph/ and select the 'Submissions Needing Revision' folder to locate your manuscript file.

Please include the following items when submitting your revised manuscript:

- A letter that responds to each point raised by the editor and reviewer(s). You should upload this letter as a separate file labeled 'Response to Reviewers'.
- A marked-up copy of your manuscript that highlights changes made to the original version. You should upload this as a separate file labeled 'Revised Manuscript with Track Changes'.
- An unmarked version of your revised paper without tracked changes. You should upload this as a separate file labeled 'Manuscript'.

Guidelines for resubmitting your figure files are available below the reviewer comments at the end of this letter.

We look forward to receiving your revised manuscript.

Kind regards,

Madhur Verma

Academic Editor

PLOS Global Public Health

Journal Requirements:

1. In the ethics statement in the Methods, you have specified that verbal consent was obtained. Please provide additional details regarding how this consent was documented and witnessed, and state whether this was approved by the IRB

2. Please provide separate figure files in .tif or .eps format.

For more information about figure files please see our guidelines:

https://journals.plos.org/globalpublichealth/s/figures

https://journals.plos.org/globalpublichealth/s/figures#loc-file-requirements

3. We have noticed that you have uploaded Supporting Information files, but you have not included a list of legends. Please add a full list of legends for your Supporting Information files after the references list.

4. In the online submission form, you indicated that “The data that support the findings of this study are available from the corresponding author upon reasonable request.”.

All PLOS journals now require all data underlying the findings described in their manuscript to be freely available to other researchers, either

1. In a public repository,

2. Within the manuscript itself, or

3. Uploaded as supplementary information.

This policy applies to all data except where public deposition would breach compliance with the protocol approved by your research ethics board. If your data cannot be made publicly available for ethical or legal reasons (e.g., public availability would compromise patient privacy), please explain your reasons by return email and your exemption request will be escalated to the editor for approval. Your exemption request will be handled independently and will not hold up the peer review process, but will need to be resolved should your manuscript be accepted for publication. One of the Editorial team will then be in touch if there are any issues.

If the reviewer comments include a recommendation to cite specific previously published works, please review and evaluate these publications to determine whether they are relevant and should be cited. There is no requirement to cite these works unless the editor has indicated otherwise.

Additional Editor Comments:

Thank you for this timely paper.

My comments:

1. The Introduction is well-written in terms of presenting the burden of NCDs and citing national datasets such as NFHS and CNNS. However, it may be helpful to strengthen the rationale slightly to more clearly justify the need for conducting this large baseline assessment.

2. At present, the paragraphs primarily describe existing prevalence data. It would add clarity if a few sentences explicitly explained how this study differs from or adds to existing datasets, particularly given that CNNS and other national surveys have already reported adolescent risk factors. Clarifying what specific gap this study addresses (for example, extension of the age range to 30 years, district-level HDSS-based profiling, integration of behavioral and biochemical measures, or linkage to the implementation arm of the V-CaN trial) would strengthen the justification.

3. Introduce your study clearly and boldly, early introduce your protocol paper and then it may be useful to state clearly that this manuscript presents baseline findings intended to inform and tailor the implementation component of the hybrid trial. Making this linkage more explicit would help readers better appreciate the added value and purpose of the baseline survey. Otherwise, readers may be high with the thought that this study would give us something from A hybrid type 2 implementation effectiveness trial, whereas, in current form it is just a cross-sectional study.

In Methods

1. I would suggest clearly adding standard subheadings for easy reading.

2. Given the number of variables assessed in this study, it may be helpful to consider presenting a consolidated table in the Methods section summarizing the operational definitions and measurement approaches used. This could include definitions of outcome variables, biochemical assessment methods (including laboratory techniques and cut-offs), procedures for estimating salt intake in grams per person per day, and the method used to assess fruit and vegetable servings and physical activity.

3. 10-20 and 20-30 years: rationale for these categories. consider aligning with The World Health Organization (WHO) or similar body, that provides specific definitions for different age groups within the young population. According to WHO, adolescents are defined as individuals between 10 and 19 years of age, young people encompass those in the age group of 10-24, and youth are categorized as those aged 15-24. Currently we are overlapping.

Results

1. Table 3: Association of stress with socio-demographic and other behavioral characteristics among 10-30-year-old persons. Why are we presenting this as a separate table, given that it was less discussed while building the rationale? Need more clarity on this. I would look for more bar charts comparing prevalence between male, female, urban/rural, and so on in a baseline paper.

2. table 4: only talks about Smokeless tobacco. rationale? We can consider tobacco as a unit, if the sample for smokers was less. is it going to change our recommendations anyways?

3. while doing regression, please recheck multicollinearity, as currently there are so many variables, that may explain causal pathways. example: role of marital status while doing regression for salt and sugar.

4. Tables in general are very cluttered, which affects readability and makes inference.

Discussion

1. Please add 4 key messages from your study, that would help u during the intervention or would inform policy, and some recommendations at the end of the discussion.

[Note: HTML markup is below. Please do not edit.]

Reviewers' comments:

Reviewer's Responses to Questions

**Comments to the Author**

1. Does this manuscript meet PLOS Global Public Health’s publication criteria?

Reviewer #1: Partly

Reviewer #2: Yes

**********

2. Has the statistical analysis been performed appropriately and rigorously?

Reviewer #1: Yes

Reviewer #2: No

**********

3. Have the authors made all data underlying the findings in their manuscript fully available (please refer to the Data Availability Statement at the start of the manuscript PDF file)?

The PLOS Data policy

Reviewer #1: Yes

Reviewer #2: No

**********

4. Is the manuscript presented in an intelligible fashion and written in standard English?

Reviewer #1: No

Reviewer #2: Yes

**********

Reviewer #1: In the abstract:

- please use the background, methods, results, conclusions subheadings.

Indicate more clearly that this is not the results of a trial, but an examination of baseline data among trial participants for a different purpose.

It is unclear whether there are 12,000 participants who provided data, or if only 1,600 provided data for analysis? How many patients in the V-CaN hybrid type 2 implementation effectiveness trial? What is the main aim of this trial and when will that be reported?

"We have also created a best-fit regression model for risk factors as outcome variables" What are the outcome variables? predictors? Please lead the reader through to understand why this was done.

- it is not clear what these abbreviations signify: RKSK, RBSK, NP-NCD, NFHS.

-was the prevalence of smoking only 0.3%?

line 34: don't use DM without explaining it.

line 35 HTN? is this the same definition of high blood pressure mentioned in line 39? If so, use same terms.

Why higher percentages of high blood pressure in 10-12 year olds compared to 13-19 year olds?

line 93: was the data cleaned using R studio? How?

line 109: (perhaps give context to 600 met-minutes - i.e. ~ an hour of running, for example, if appropriate.

line 120: Capitals for American Academy of Pediatrics.

Table 1: is Other backward class the best descriptor?

Could there be a figure to show the predictors of each outcome?

line 227: should be 0.50

line 227: compared to a farmer and laborer

Instead of gender, should it be sex?

Were the blood samples only tested for blood glucose status?

Reviewer #2: Overall relevance and significance

The topic is highly relevant, as studying NCD risk factors among adolescents is important because this age group is particularly vulnerable to commercial determinants of health.

Adolescence represents a critical window for early prevention, making the study timely and valuable.

1. Clarification on participant flow

Please clarify what happened to participants who consented for testing but were not included.

You mention that ~11,000 agreed, but the final sample was 1,600.

A clear flow diagram (screened → consented → included → analyzed) with reasons for exclusion would improve transparency and reduce potential selection bias concerns.

2. Regression modelling approach

In the prediction models, consider including only biologically plausible predictors for each outcome.

For example, for stress, variables such as psychosocial factors may be more appropriate.

For salt intake, predictors like dietary patterns or processed food consumption would be more relevant.

Some associations (e.g., smoking with higher salt intake, or stress with vegetable intake) may not be conceptually necessary and could introduce overadjustment or model instability.

3. Variable selection strategy

Instead of stepwise methods, an enter (forced entry) method based on conceptual framework is preferable.

This helps avoid data-driven selection and preserves meaningful associations grounded in theory.

4. Interpretation of findings

The discussion should expand on the biological and behavioral plausibility of key associations.

For example, explain why salt intake might relate to physical activity (e.g., dietary patterns, sports drinks, higher caloric intake).

Similar explanatory reasoning with supporting literature should be provided for other significant findings.

5. Strengthening the discussion

Link findings more explicitly to existing evidence and mechanisms, not just statistical significance.

Highlight how results inform adolescent NCD prevention strategies or policy implications.

**********

what does this mean?). If published, this will include your full peer review and any attached files.

**Do you want your identity to be public for this peer review?**  If you choose “no”, your identity will remain anonymous but your review may still be made public.

For information about this choice, including consent withdrawal, please see our Privacy Policy

Reviewer #1: No

Reviewer #2: **Yes:**  Sumanth Mallikarjuna Majgi

**********

[NOTE: If reviewer comments were submitted as an attachment file, they will be attached to this email and accessible via the submission site. Please log into your account, locate the manuscript record, and check for the action link "View Attachments". If this link does not appear, there are no attachment files.]

# Author comment: Author response to Decision Letter 1

**DOI:** 10.1371/journal.pgph.0005956.r003

17 Apr 2026

Attachment Submitted filename: PLOS Global Public Health_response to reviewers.docx

# Peer review: Decision Letter 1

**DOI:** 10.1371/journal.pgph.0005956.r004

**Authors:** Jen Edwards

10 Aug 2026

PGPH-D-25-03509R1

Prevalence and predictors of risk factors for non-communicable diseases in individuals aged 10-30 years: Findings from baseline assessment of V-CaN – A hybrid type 2 implementation effectiveness trial

PLOS Global Public Health

Dear Dr. Raut,

Thank you for submitting your manuscript to PLOS Global Public Health. After careful consideration, we feel that it has merit but does not fully meet PLOS Global Public Health’s publication criteria as it currently stands. Therefore, we invite you to submit a revised version of the manuscript that addresses the points raised during the review process.

The manuscript has been evaluated by two reviewers and their comments are available below. The reviewers have raised a number of concerns that need attention. They request a number of clarifications regarding the consistent use of terminology and improvement in the quality of language. In addition, they express concerns over the appropriateness of the regression analysis conducted. Could you please revise the manuscript to carefully address the concerns raised?

Please submit your revised manuscript by Oct 06 2026 11:59PM. If you will need more time than this to complete your revisions, please reply to this message or contact the journal office at globalpubhealth@plos.org. When you're ready to submit your revision, log on to https://www.editorialmanager.com/pgph/ and select the 'Submissions Needing Revision' folder to locate your manuscript file.

Please include the following items when submitting your revised manuscript:

- A letter that responds to each point raised by the editor and reviewer(s). You should upload this letter as a separate file labeled 'Response to Reviewers'.
- A marked-up copy of your manuscript that highlights changes made to the original version. You should upload this as a separate file labeled 'Revised Manuscript with Track Changes'.
- An unmarked version of your revised paper without tracked changes. You should upload this as a separate file labeled 'Manuscript'.

Guidelines for resubmitting your figure files are available below the reviewer comments at the end of this letter.

As the corresponding author, your ORCID iD is verified in the submission system and will appear in the published article. PLOS supports the use of ORCID, and we encourage all coauthors to register for an ORCID iD and use it as well. Please encourage your coauthors to verify their ORCID iD within the submission system before final acceptance, as unverified ORCID iDs will not appear in the published article. *Only*  the individual author can complete the verification step; PLOS staff *cannot*  verify ORCID iDs on behalf of authors.

We look forward to receiving your revised manuscript.

Kind regards,

Jen Edwards

Staff Editor

PLOS Global Public Health

**Journal Requirements:**

If the reviewer comments include a recommendation to cite specific previously published works, please review and evaluate these publications to determine whether they are relevant and should be cited. There is no requirement to cite these works unless the editor has indicated otherwise.

**Additional Editor Comments (if provided):**

[Note: HTML markup is below. Please do not edit.]

Reviewers' comments:

Reviewer's Responses to Questions

**Comments to the Author**

Reviewer #1: All comments have been addressed

Reviewer #3: (No Response)

Reviewer #4: All comments have been addressed

**********

publication criteria?

Reviewer #1: (No Response)

Reviewer #3: No

Reviewer #4: Yes

**********

3. Has the statistical analysis been performed appropriately and rigorously?

Reviewer #1: (No Response)

Reviewer #3: No

Reviewer #4: Yes

**********

4. Have the authors made all data underlying the findings in their manuscript fully available (please refer to the Data Availability Statement at the start of the manuscript PDF file)?

The PLOS Data policy

Reviewer #1: (No Response)

Reviewer #3: No

Reviewer #4: Yes

**********

5. Is the manuscript presented in an intelligible fashion and written in standard English?

Reviewer #1: (No Response)

Reviewer #3: No

Reviewer #4: Yes

**********

**Reviewer #1:**  (No Response)

**Reviewer #3:**  Kalantri et al. submitted a manuscript titled “Prevalence and predictors of risk factors for non-communicable diseases in individuals aged 10–30 years: Findings from baseline assessment of V CaN – A hybrid type 2 implementation effectiveness trial.” While the research topic is important and has the potential to contribute meaningfully to the literature, the manuscript has substantial methodological and scientific shortcomings. Issues span from the description of the sample size calculation to the analytical approach, and the overall reporting lacks scientific rigor. In addition, several sections are unclear, inconsistent, or do not adhere to established reporting standards such as STROBE.

Major Concerns:

1) Title, study design, and objectives: The manuscript presents conflicting descriptions of the study design and objectives. The title refers to an “implementation effectiveness trial,” the introduction describes the work as “implementation research,” the methods section labels it a “cross-sectional survey,” and the authors’ response to previous reviewer comments states that the analysis is based on baseline data from trial participants. These inconsistencies undermine the clarity and coherence of the study. Similar inconsistencies appear throughout the manuscript and make the study not scientifically sound.

2) Sample size calculation: The stated aim is to estimate prevalence; however, the sample size calculation does not follow standard methods for prevalence estimation. For prevalence studies, the recommended approach is the formula z^2 pq/d^2, which appropriately accounts for the expected prevalence and desired precision. The current method is not aligned with accepted epidemiologic practice.

3) Analytical approach: The regression analyses appear to be entirely data driven, with no underlying causal framework or theoretical justification guiding variable selection. In epidemiologic research, risk factor analyses require a clear causal rationale to avoid biased or uninterpretable estimates. Without incorporating subject matter knowledge or causal structure (e.g., directed acyclic graphs, conceptual models), the current models are scientifically inappropriate and may yield misleading results.

4) Overall writing: The overall writing quality is insufficient for publication. Several sections are unclear, contain errors, or lack the precision expected in scientific manuscripts.

**Reviewer #4:**  I would like to thank the authors for the opportunity to review this manuscript. The study addresses an important and relevant topic and has the potential to contribute meaningfully to the existing literature.

The manuscript is generally well organized, and the objectives are clearly stated. The methodology appears appropriate for the research question, and the results are presented in a logical manner. However, several areas would benefit from further clarification and revision to improve the overall quality and readability of the manuscript. I did not identify any major concerns regarding research ethics or publication ethics based on the information provided. Subject to satisfactory revision of the points noted above, the manuscript may be suitable for publication.

**********

what does this mean?). If published, this will include your full peer review and any attached files.

**Do you want your identity to be public for this peer review?**  If you choose “no”, your identity will remain anonymous but your review may still be made public.

For information about this choice, including consent withdrawal, please see our Privacy Policy

Reviewer #1: No

Reviewer #3: No

Reviewer #4: **Yes:**  Dr. Rabia Zulfiqar (Worldwide Record Holder in Research Certifications) rabiazulfiqar@outlook.com

**********

[NOTE: If reviewer comments were submitted as an attachment file, they will be attached to this email and accessible via the submission site. Please log into your account, locate the manuscript record, and check for the action link "View Attachments". If this link does not appear, there are no attachment files.]

**Figure Resubmissions:**

# Author comment: Author response to Decision Letter 2

**DOI:** 10.1371/journal.pgph.0005956.r005

7 Sep 2026

Attachment Submitted filename: VCaN response2.docx

# Peer review: Decision Letter 2

**DOI:** 10.1371/journal.pgph.0005956.r006

**Authors:** Julia Robinson

28 Sep 2026

Prevalence and predictors of risk factors for non-communicable diseases in individuals aged 10-30 years: Findings from baseline cross-sectional assessment of V-CaN

PGPH-D-25-03509R2

Dear Dr Raut,

We are pleased to inform you that your manuscript 'Prevalence and predictors of risk factors for non-communicable diseases in individuals aged 10-30 years: Findings from baseline cross-sectional assessment of V-CaN ' has been provisionally accepted for publication in PLOS Global Public Health.

Before your manuscript can be formally accepted you will need to complete some formatting changes, which you will receive in a follow up email. A member of our team will be in touch with a set of requests.

Please note that your manuscript will not be scheduled for publication until you have made the required changes, so a swift response is appreciated.

IMPORTANT: The editorial review process is now complete. PLOS will only permit corrections to spelling, formatting or significant scientific errors from this point onwards. Requests for major changes, or any which affect the scientific understanding of your work, will cause delays to the publication date of your manuscript.

If your institution or institutions have a press office, please notify them about your upcoming paper to help maximize its impact. If they'll be preparing press materials, please inform our press team as soon as possible -- no later than 48 hours after receiving the formal acceptance. Your manuscript will remain under strict press embargo until 2 pm Eastern Time on the date of publication. For more information, please contact globalpubhealth@plos.org.

Thank you again for supporting Open Access publishing; we are looking forward to publishing your work in PLOS Global Public Health.

Best regards,

Julia Robinson

Executive Editor

PLOS Global Public Health

***********************************************************

Reviewer Comments (if any, and for reference):

Reviewer's Responses to Questions

**Comments to the Author**

Reviewer #1: All comments have been addressed

**********

publication criteria?

Reviewer #1: (No Response)

**********

3. Has the statistical analysis been performed appropriately and rigorously?

Reviewer #1: (No Response)

**********

4. Have the authors made all data underlying the findings in their manuscript fully available (please refer to the Data Availability Statement at the start of the manuscript PDF file)?

The PLOS Data policy

Reviewer #1: (No Response)

**********

5. Is the manuscript presented in an intelligible fashion and written in standard English?

Reviewer #1: (No Response)

**********

Reviewer #1: (No Response)

**********

what does this mean?). If published, this will include your full peer review and any attached files.

**Do you want your identity to be public for this peer review?**  If you choose “no”, your identity will remain anonymous but your review may still be made public.

For information about this choice, including consent withdrawal, please see our Privacy Policy

Reviewer #1: No

**********
